# The amyloid-inhibiting NCAM-PrP peptide targets Aβ peptide aggregation in membrane-mimetic environments

**DOI:** 10.1016/j.isci.2021.102852

**Published:** 2021-07-10

**Authors:** Sylwia Król, Nicklas Österlund, Faraz Vosough, Jüri Jarvet, Sebastian Wärmländer, Andreas Barth, Leopold L. Ilag, Mazin Magzoub, Astrid Gräslund, Cecilia Mörman

**Affiliations:** 1 Department of Biochemistry and Biophysics, Stockholm University, Stockholm, 106 91, Sweden; 2 Department of Materials and Environmental Chemistry, Stockholm University, Stockholm, 106 91, Sweden; 3 Biology Program, Division of Science, New York University Abu Dhabi, Box 129188, Abu Dhabi, United Arab Emirates

**Keywords:** molecular neuroscience, structural biology, biophysics

## Abstract

Substantial research efforts have gone into elucidating the role of protein misfolding and self-assembly in the onset and progression of Alzheimer’s disease (AD). Aggregation of the Amyloid-β (Aβ) peptide into insoluble fibrils is closely associated with AD. Here, we use biophysical techniques to study a peptide-based approach to target Aβ amyloid aggregation. A peptide construct, NCAM-PrP, consists of a largely hydrophobic signal sequence linked to a positively charged hexapeptide. The NCAM-PrP peptide inhibits Aβ amyloid formation by forming aggregates which are unavailable for further amyloid aggregation. In a membrane-mimetic environment, Aβ and NCAM-PrP form specific heterooligomeric complexes, which are of lower aggregation states compared to Aβ homooligomers. The Aβ:NCAM-PrP interaction appears to take place on different aggregation states depending on the absence or presence of a membrane-mimicking environment. These insights can be useful for the development of potential future therapeutic strategies targeting Aβ at several aggregation states.

## Highlights

• A signal peptide construct, NCAM-PrP, inhibits Aβ peptide amyloid aggregation
• Aβ and NCAM-PrP form co-aggregates which are not compatible with amyloid formation
• The Aβ and NCAM-PrP interaction occurs both in aqueous solution and in membranes
• Co-aggregates formed in solution and in the membrane have different properties

## Graphical abstract

**Figure:**
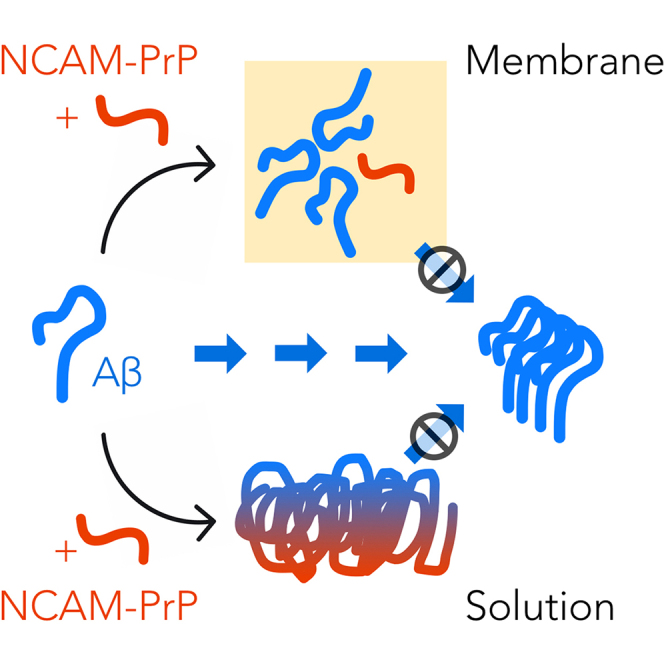

## Introduction

The Amyloid-β (Aβ) peptide, 40 or 42 residues long, is one of the major actors in the neurotoxic mechanisms involved in Alzheimer’s disease (AD). AD is a major human neurodegenerative disease, today affecting more than 20-40 million people in the world (Mayeux and Stern 2012; Guerchet et al., 2013). No efficient therapy has been found, despite numerous therapeutic and clinical strategies (Ankarcrona et al., 2016).

The biological processes leading to the disease have been elucidated to some extent in the past 30 years, but many unsolved questions remain. Among the unresolved issues is the lack of consensus on where the important disease processes take place (i.e., outside or within neurons) and how they proceed. The so-called amyloid cascade hypothesis (ACH) (Hardy and Higgins 1992), based on aggregation of the Aβ peptide into amyloid aggregates, has been generally accepted (Selkoe and Hardy 2016). The amyloid formation involves Aβ as a main actor, and this primary process is considered to be linked to secondary processes such as brain inflammation (Kinney et al., 2018), leading to neuronal cell death. Phospholipid membranes are generally considered to be important for Aβ amyloid formation. *In vitro*, lipid vesicles and various membrane-mimetics have been shown to affect the amyloid process, including intermediate molecular structures and aggregation kinetics (Kotler et al., 2014; Österlund et al., 2019; Butterfield and Lashuel 2010). Despite many studies, the molecular details of this phenomenon are still unclear. Several mechanisms by which the membrane affects the self-assembly of Aβ have been suggested. Membrane models of zwitterionic dioleoyl-phosphatidylcholine (DOPC) lipid vesicles have been shown to both enhance and suppress amyloid formation dependent on the experimental conditions such as shaking or quiescent conditions, respectively (Hellstrand et al., 2010; Lindberg et al., 2017). Interactions between protein aggregates and membranes are also suggested to compromise the integrity of a cellular membrane, with possible adverse effects on cell viability (Ke et al., 2020; Andreasen et al., 2015). Moreover, mechanisms by which the membrane is ruptured by growing amyloid fibrils have been suggested (Ke et al., 2020). Cell homeostasis could also be disturbed by formation of small oligomeric Aβ membrane pores, or by thinning or curving of the membrane bilayer by Aβ monomers and/or oligomers (Ke et al., 2020; Ciudad et al., 2020; Quist et al., 2005). Several of these mechanisms could likely co-exist both *in vitro* and *in vivo* and could be modulated by specific experimental or cellular conditions such as peptide assembly state, bilayer composition, interaction partners, pH, or ionic strength.

The molecular details of Aβ amyloid aggregation have been studied using a variety of (bio)chemical and biophysical methods (Cremades and Dobson 2018; Wallin et al., 2017). A fluorescence probe, ThioflavinT (ThT), binds to the β-sheet rich structure of amyloid fibrils, whereby its fluorescence yield increases significantly (Biancalana and Koide 2010; Gade Malmos et al., 2017). ThT is therefore often used to follow the kinetics of the amyloid formation process. After an initial so-called “lag phase” when smaller prefibrillar Aβ aggregates are formed, secondary self-catalyzed reactions lead to amyloid fibrillar formation in a “transition phase”, until the process is complete (Dobson 2004; Meisl et al., 2018; Arosio et al., 2016). The dominating process of Aβ fibrillization is monomer-dependent secondary nucleation (Meisl et al., 2014; Cohen et al., 2013). Recent studies indicate that it is mainly the protofibrillar Aβ aggregates that are most toxic to the neuronal cells (Dubnovitsky et al., 2013; Sengupta et al., 2016; Haass and Selkoe 2007). Such structures have recently been found to mostly be generated by fibril dependent secondary nucleation processes *in vitro* (Michaels et al., 2020). What is not well known is where the most toxic aggregates are formed and/or transported *in vivo*, and where these aggregates induce their toxicity – inside or outside the cells, or both.

The present study focuses on the Aβ amyloid formation process *in vitro* and how it is modulated by a designed anti-amyloid peptide with a signal sequence motif. This peptide is part of a group of peptides originally defined as anti-prion peptides, since they could inhibit an ongoing prion propagation/conversion in prion-infected neuronal cells (Söderberg et al., 2014). Notably, these peptides have also been shown to act as cell penetrating peptides (CPPs) (Lundberg et al., 2002).

Recently, we showed that such CPPs can effectively antagonize cytotoxicity caused by exposure of neuroblastoma cells to exogenously added Aβ (Henning-Knechtel et al., 2020). The original anti-prion CPP was composed of residues 1–28 of the mouse prion protein (mPrP) or residues 1–30 of the bovine prion protein (bPrP), with a generally hydrophobic signal sequence (mPrP_1-22_) followed by a largely cationic hexameric sequence/segment (KKRPKP, mPrP_23-28_) after the signal peptidase cleavage site (Figure S1A) (Söderberg et al., 2014). Subsequently, we determined that the prion signal sequence could be replaced with a shorter and less hydrophobic signal sequence from the NCAM1 protein (NCAM1_1-19_) with equal efficiency against prion conversion when coupled with mPrP_23-28_ (Söderberg et al., 2014). The NCAM1 protein has been suggested as an interaction partner of both the prion protein and the Aβ precursor protein (AβPP) (Schmitt-Ulms et al., 2001; Santuccione et al., 2005; Leshchyns’Ka and Sytnyk 2016). This NCAM1_1-19_-mPrP_23-28_ construct (Figure S1B) is the topic of this study and will here be referred to as NCAM-PrP. It has, however, also been found that the C-terminal hexapeptide (mPrP_23-28_) can be replaced by the KKLVFF hexapeptide (KLVFF originating from the Aβ peptide) in this NCAM1-construct without loss of activity (Henning-Knechtel et al., 2020). Two relatively new aspects of cell toxicity involving the Aβ peptide are important here: The Aβ toxic amyloid aggregates may be formed at least partly inside cells, and they can be transported between cells, thereby spreading the AD toxicity, similar to prion toxicity (Domert et al., 2014; Ji et al., 2016; LaFerla et al., 2007; Sakono and Zako 2010).

Here, we present results of further *in vitro* studies on how this anti-amyloid CPP (NCAM1_1-19_-mPrP_23-28_, see Methods details for the primary sequence) interacts with the 42 and 40 residues long Aβ peptides, as monomers and while undergoing the amyloid aggregation process. In particular, we have investigated the potential role of membrane interactions for the anti-amyloid effects. Our hypothesis is that the two peptides may interact outside or inside neurons, as well as possibly on/within the neuronal plasma- and/or intracellular organelle membranes.

## Results

### LUV leakage experiments induced by NCAM-PrP

**Figure 1:**
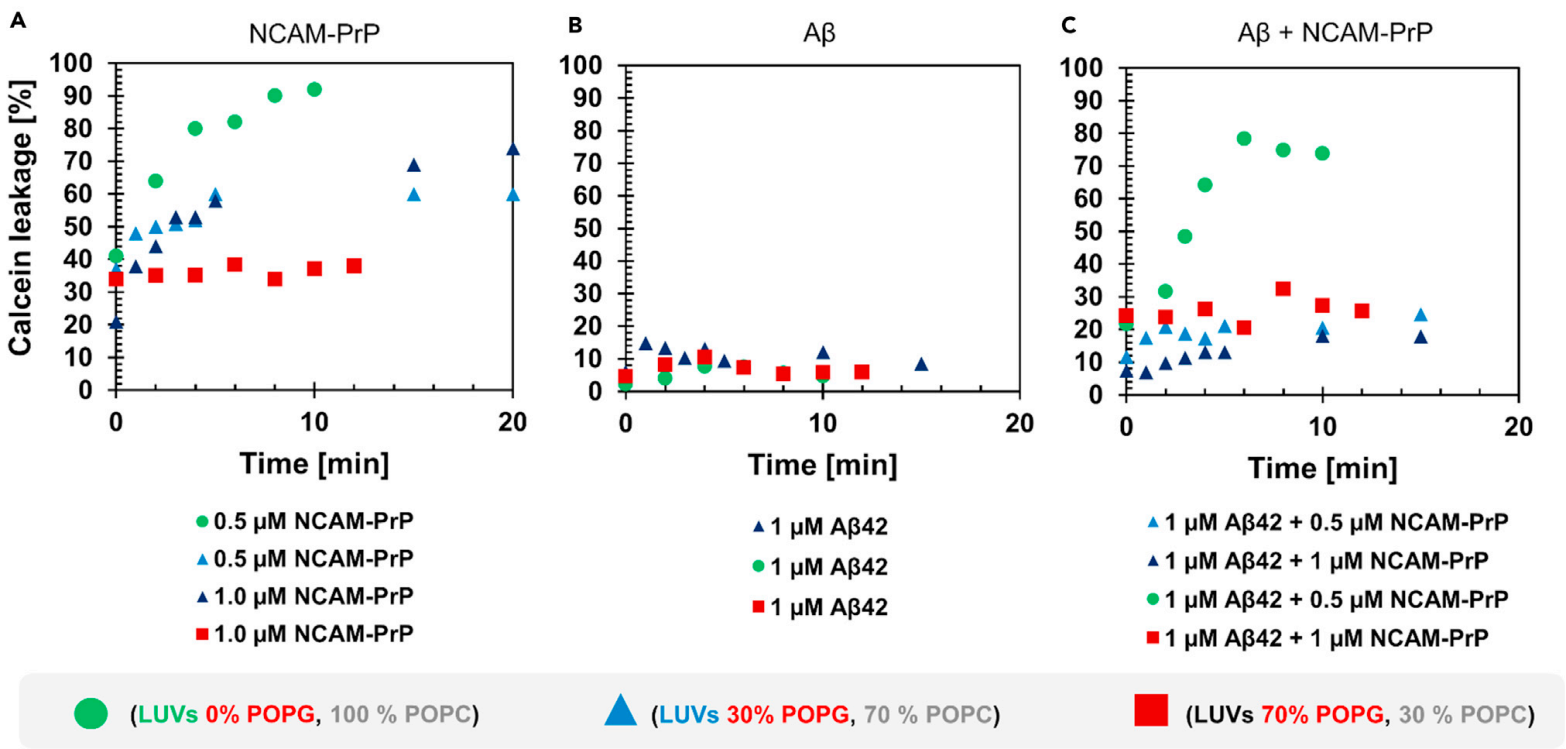
NCAM-PrP perturbs large unilamellar vesicles (LUVs) and causes calcein leakage Time-dependence of the calcein leakage in % of added (A) NCAM-PrP or (B) Aβ_42_ to different compositions (surface charge) of LUVs. In (C) Aβ_42_ and NCAM-PrP were simultaneously added to the LUVs. All experiments were carried out in 50 mM potassium phosphate buffer pH 7.4 at +5°C. Green circles correspond to zwitterionic LUVs with 100% POPC lipid composition, whereas blue triangles represent negatively charged LUVs with 30% POPG and 70% POPC lipids, and highly negatively charged LUVs are presented as red squares with 70% POPG and 30% POPC lipid composition. 55 mM calcein was entrapped inside the LUVs. One single measurement per condition was measured. See also Figure S1.

Calcein-encapsulated large unilamellar vesicles (LUVs) were used as a simple, biophysical membrane-mimetic to study the capacity of NCAM-PrP and Aβ_42_ to perturb lipid membranes. The lipid compositions of the LUVs were varied to model eukaryotic (zwitterionic) and prokaryotic (negatively charged) lipid membranes. LUVs with three different surface charge densities, formed by varying the content of negatively charged POPG and neutral POPC lipids, were used. The high concentration of calcein entrapped within the lumen of the LUVs is characterized by a low fluorescence intensity level due to self-quenching of the dye. Perturbation of the LUV lipid membrane leads to release of calcein into the surrounding medium and dilution of the dye’s concentration, resulting in a rapid and substantial increase in the fluorescence intensity (Andersson et al., 2007; Zhang et al., 2010; Madani and Gräslund 2015). In Figure 1A, the release of calcein from addition of freshly prepared monomeric NCAM-PrP was monitored over time. Depending on the lipid composition of the LUVs, different rates of calcein release were observed. In zwitterionic (100% POPC) LUVs, the leakage efficiency of calcein was high and reached almost 100% leakage after 10 min. Introducing negatively charged lipids to the LUVs (30% POPG, 70% POPC) reduced the leakage efficiency, reaching approximately 60-70% leakage after 20 min. The shapes of the calcein leakage curves are biphasic, with a fast and exponential increase followed by a slower, more gradual release. This behavior has also been observed for other peptides able to perturb lipid membranes (Zhang et al., 2010). For highly negatively charged LUVs (70% POPG, 30% POPC), no leakage was observed following addition of peptide (Figure 1A).

The leakage efficiencies of Aβ_42_ alone and for the Aβ_42_:NCAM-PrP complex in LUVs were also investigated (Figures 1B and 1C). Under our experimental conditions, additions of monomeric Aβ_42_ to zwitterionic or negatively charged LUVs only induced minor leakage, around 10% (Figure 1B). As a control, the leakage efficiency of aged Aβ_42_ samples was also investigated and no significant calcein leakage was detected (data not shown). Interestingly, when the two peptides were studied together, Aβ_42_ reduced the NCAM-PrP leakage efficiency in zwitterionic (100% POPC) LUVs from about 100% (Figure 1A) to approximately 80% (Figure 1C). In negatively charged LUVs (30% POPG, 70% POPC), the NCAM-PrP-induced calcein leakage was decreased to about 20% in the presence of Aβ_42_, compared to around 60-70% leakage in the absence of Aβ_42_. The reduction of NCAM-PrP-induced leakage by the presence of monomeric Aβ_42_ is likely due to the Aβ_42_:NCAM-PrP interaction (Figure 1C).

### NCAM-PrP attenuates the formation of Aβ amyloid fibrils

#### Circular dichroism (CD) spectroscopy to study the changes in secondary structures over time

**Figure 2:**
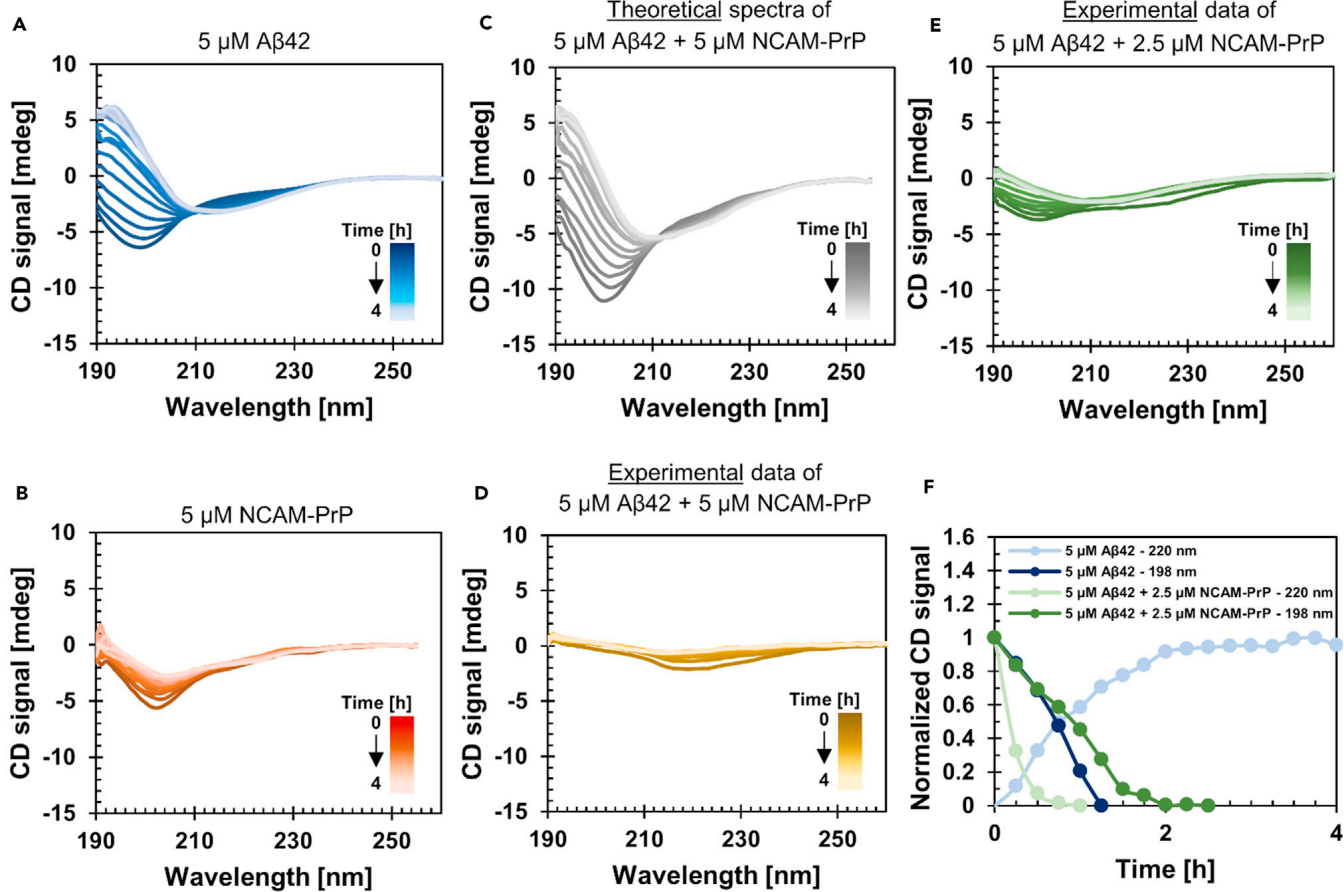
Circular dichroism (CD) spectroscopy to study the secondary structures of Aβ_42_ and NCAM-PrP peptides over time (A) 5 μM Aβ_42_ peptides incubated over time (0–4 h) in 10 mM sodium phosphate (NaP) buffer pH 7.3 at +37°C with constant stirring using a small magnet. (B) Incubation of 5 μM NCAM-PrP peptides in 10 mM NaP buffer pH 7.3 at +37°C with magnetic stirring. If the spectra in (A) and (B) are added together at each time point a theoretical presentation of how the spectra would look like if the two peptides are co-incubated together without any interaction is achieved, and this presentation is presented in (C). However, if the two peptides (5 μM Aβ_42_ and 5 μM NCAM-PrP) are experimentally co-incubated together (starting with monomeric samples) the CD spectra show a completely different behavior (D) compared to the theoretical one in (C). This observation suggests that the two peptides interact. A kinetic experiment of 5 μM Aβ_42_ in the presence of 2.5 μM NCAM-PrP is presented in (E). In (F) are the CD signals at 198 nm (random coil) and 220 nm (β-structures) from the spectra for 5 μM Aβ_42_ alone in the presence of 2.5 μM NCAM-PrP normalized and presented together. See also Figures S2–S5.

**Figure 3:**
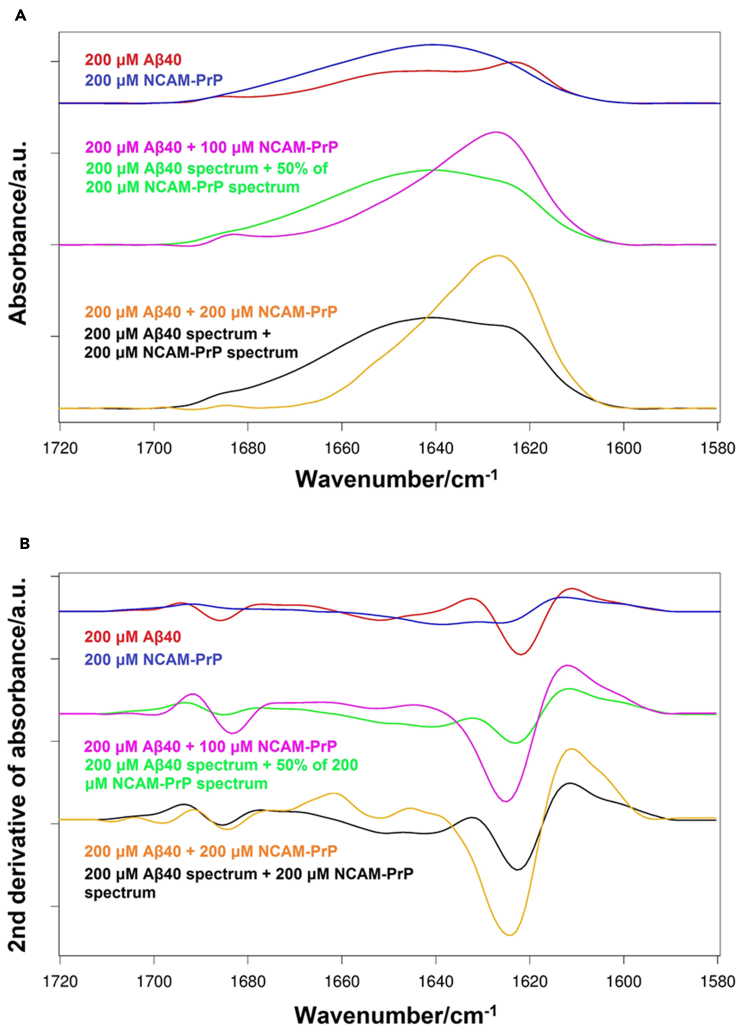
Absorption and second derivative IR spectra of the NCAM-PrP peptide and Aβ_40_-NCAM-PrP complex IR measurements were performed in liquid of 200 μM Aβ_40_ in 20 mM NaP buffer at physiological pH (D_2_O) alone and together with 100 or 200 μM NCAM-PrP in 10 mM NaP buffer pH 7.3, presented as absorption spectra (A) and as the second derivative (B).

To investigate how the secondary structures of the Aβ_40_ and Aβ_42_ peptides are affected in the presence of NCAM-PrP, several CD spectra were recorded over time. A monomeric Aβ_42_ peptide sample exhibits mainly disordered and random coil secondary structure as observed by CD spectra with a negative signal at 198 nm, but over time the Aβ_42_ aggregates into β-sheet secondary structures with a minimum around 218-220 nm and a maximum around 195 nm. 5 μM Aβ_42_ peptides transitioned into a mostly β-sheet state within 4 h, under agitation with stirring (Figure 2A). In comparison, 5 μM NCAM-PrP did not form β-sheet secondary structures over time under similar experimental conditions (Figure 2B). However, at higher NCAM-PrP peptide concentration there are β-sheet structures partly present, for 20 μM NCAM-PrP in the CD spectrum (Figure S2) as well as in the FTIR spectrum at a 200 μM concentration (Figure 3). Theoretical spectra for mixtures of the two peptides were generated by adding the signal intensities of the spectra of each individual peptide at each time point (Figure 2C). Such a theoretical spectrum illustrates how an experimental spectrum of the peptide mixture would look like if there were no interactions between the two peptides that change either of the peptide’s secondary structures. The Aβ_42_ and NCAM-PrP peptides were also experimentally co-incubated over time (Figure 2D). In contrast to the theoretical spectra in Figure 2C, the experimental data shows a completely different behavior (Figures 2D and S3). The CD signals at 198 nm are completely abolished, either by no presence of random coil structures or by interference with positive signals. A low signal of β-sheet structures is present at time zero, and over time this signal approaches zero. Loss of signal may be explained by formation of larger structures which are not observable by CD spectroscopy. The kinetic experiment with Aβ_42_ was also repeated with half the concentration of NCAM-PrP (Figure 2E), where CD signals for both random coil and β-structures were present before a gradual loss of signal over time. To test the stability of the Aβ:NCAM-PrP complexes, the samples in Figures 2A and 2E were treated with SDS micelles after the kinetic experiment, and α-helical structures were instantly induced (Figure S4).

To compare the Aβ_42_ aggregation behavior in the absence and presence of NCAM-PrP (Figures 2A and 2E), the CD signals at 198 nm and 220 nm were normalized and plotted together in Figure 2F. For Aβ_42_ alone, at 220 nm the signal intensity increases over time and when normalized the data curve goes from zero towards one. Conversely, the CD signal at 198 nm, corresponding to random coil structure, decreases over time and is characterized with a sigmoidal behavior from one toward zero. The two curves for Aβ_42_ in Figure 2F meet after approximately one hour of incubation, corresponding to the isodichroic point in Figure 2A. In contrast, when Aβ_42_ is co-incubated with the NCAM-PrP peptide the normalized data curves exhibit another appearance. The CD signal at 198 nm shows a similar behavior as in the absence of NCAM-PrP peptide, but the CD signal at 220 nm is completely different. Instead of an increased amount of β-sheet structure, the presence of β-sheet secondary structure content is decreased in the presence of NCAM-PrP.

The Aβ_40_ peptide variant displays a similar behavior in the presence of NCAM-PrP peptides (Figure 3 and Figure S5) as the Aβ_42_ peptide. Co-incubation of Aβ_40_/Aβ_42_ and NCAM-PrP clearly affects the aggregation kinetics of Aβ_40_/Aβ_42_ with less signal intensity for β-sheet secondary structures left.

#### FTIR spectroscopy of the Aβ_40_-NCAM-PrP interaction

Absorption and second derivative IR spectra for pure Aβ_40_, pure NCAM-PrP and mixtures of the two peptides with molar ratios of 2:1 and 1:1 are shown in Figures 3A and 3B. The absorbance spectra were normalized to the same integrated amide I area per amide group. The second derivative spectrum for pure Aβ_40_ (panel B, blue) shows two major bands in the amide I region (1700-1600 cm^−1^) at 1638 cm^−1^ and 1626 cm^−1^, which indicates that the peptide's secondary structure at the beginning of aggregation at pD 7.4 is comprised of both random coil and β-sheet conformations. The second derivative spectrum for NCAM-PrP (red) has a major band at about 1622 cm^−1^ and a small high wavenumber band at 1686 cm^−1^, denoting the presence of anti-parallel β-sheet structure of the peptide in phosphate buffer, pD 7.4. A broad band is also observed around 1650 cm^−1^, which implies that other secondary structure elements (possibly helices) also contribute to the structure of the peptide.

In the middle of each panel, the IR spectra for the mixture of 200 μM Aβ_40_ and 100 μM NCAM-PrP are displayed. The experimental spectrum is compared to a hypothetical spectrum (green) calculated by adding the 200 μM Aβ_40_ IR spectrum and 50% of the 200 μM NCAM-PrP IR spectrum. This spectrum is expected when the conformation of the pure compounds is preserved in the mixture. However, the calculated and experimental spectra are clearly different, indicating an interaction between the two peptides. The intensity of the band at 1625 cm^−1^ is higher in the spectrum recorded from the experiment and also a high wavenumber band at about 1685 cm^−1^ is detected, traits which are not easily distinguished in the artificial spectrum. Moreover, the band intensity in the center of the amide I region is reduced in the experimental spectrum. Put together, such spectral characteristics confirm the formation of more β-sheet secondary structure when peptides interact in phosphate buffer, pD 7.4. The presence of the small band at 1685 cm^−1^ indicates that the formed β-sheet structure maintains an at least partially antiparallel conformation. At the bottom of each panel, IR spectra for a 1:1 mixture of the two peptides are shown. Again, the spectrum from the experiment (orange) is compared to the artificial spectrum (black) prepared by adding up the IR spectra for 200 μM solutions of Aβ_40_ and NCAM-PrP. Similar to the sample of 2:1 molar ratio, the experimental spectrum points to a higher β-sheet content in comparison to the sum of the spectra for the two peptides.

The intensity of the high wavenumber band at 1685 cm^−1^ appears to be reduced in the Aβ_40_:NCAM-PrP 1:1 mixture relative to the 2:1 mixture. In a typical Aβ peptide aggregation experiment in neutral pD studied by IR spectroscopy, the intensity of the high wavenumber band increases over time for a couple of hours, which indicates formation of soluble aggregates of predominantly anti-parallel structure (Vosough and Barth 2021). In this context, the reduction of intensity for the high wavenumber band after mixing Aβ_40_ and NCAM-PrP peptides is notable, since it suggests a shift toward parallel β-sheet secondary structure in the 1:1 mixture.

#### The NCAM-PrP retardation effect on the Aβ amyloid formation is not affected by increased ionic strength or by addition of pre-formed seeds

**Figure 4:**
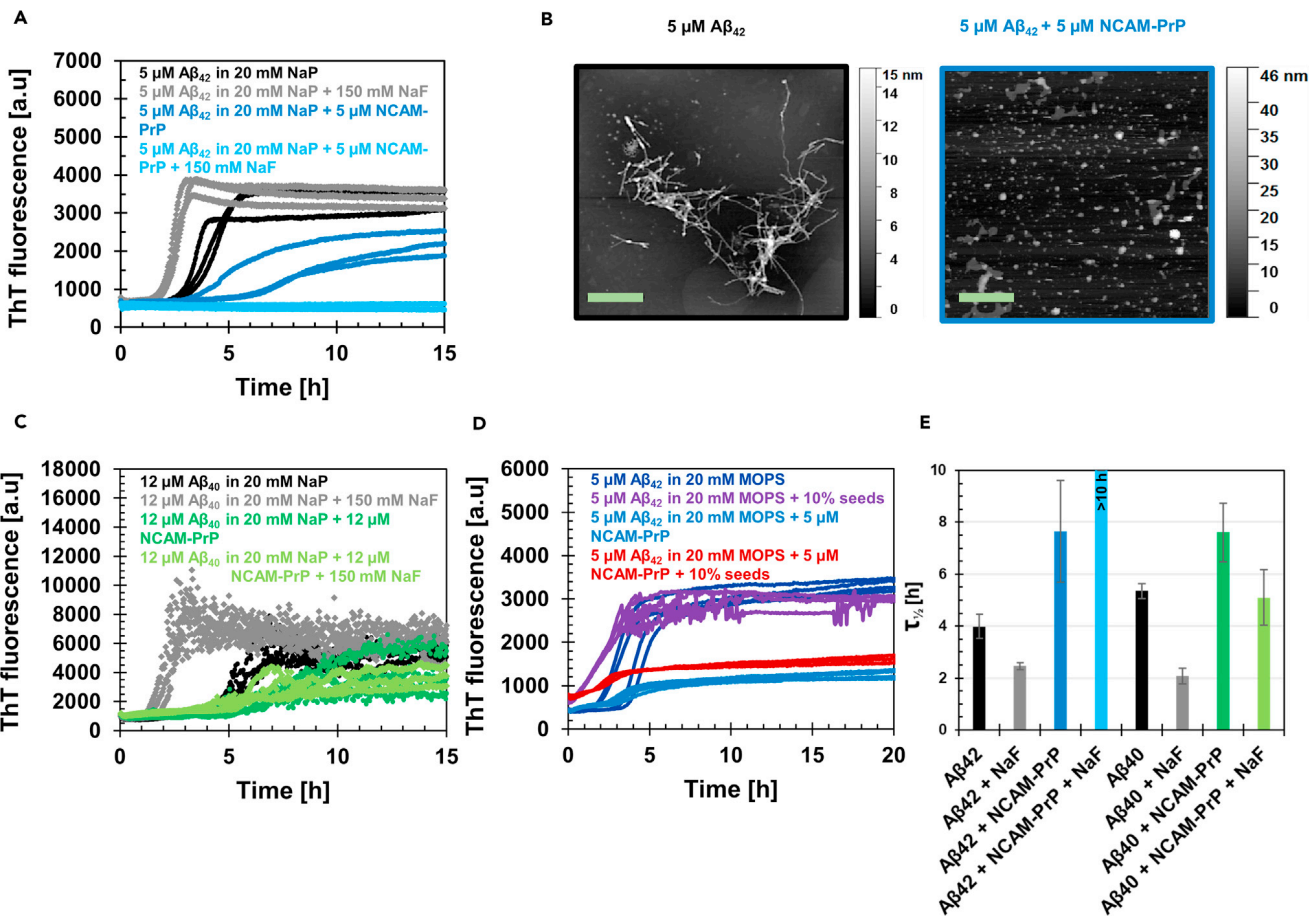
Thioflavin T (ThT) kinetics assay and atomic force microscopy (AFM) to study how monomeric NCAM-PrP peptides affect the Aβ_40_ and Aβ_42_ amyloid formation under different conditions (A) 5 μM Aβ_42_ monomeric peptides incubated in the presence and absence of 5 μM monomeric NCAM-PrP peptides, with/without 150 mM NaF, were monitored by ThT fluorescence intensity measurements over time under quiescent conditions. 20 mM sodium phosphate (NaP) buffer pH 8 and 10 μM ThT was used. (B) At the end of the ThT kinetic assay in (A) samples were taken and put on mica surfaces and investigated with AFM imaging. For Aβ_42_ peptide alone (black) typical amyloid fibrils were observed, whereas for Aβ_42_ peptides incubated in the presence of NCAM-PrP peptides no fibrils were detected (dark blue). The scale bar corresponds to 1 μm. (C) The experiments in (A) were also performed with the Aβ_40_ peptide variant. 12 μM Aβ_40_ peptide, 12 μM NCAM-PrP, 20 mM NaP buffer pH 7.3, quiescent conditions and 40 μM ThT was used. (D) Seeding experiments were performed on the Aβ_42_ peptide (5 μM monomeric Aβ_42_) variant using 5 μM NCAM-PrP and 10% Aβ_42_ pre-formed fibrils in MOPS buffer pH 7.8. (E) Aggregation half-time (τ_½_) of the kinetic curves in (A,C). For the sample with Aβ_42_ + NCAM-PrP + NaF the amyloid formation was completely inhibited with no detected increase in ThT fluorescence intensity signal, therefore τ_½_ is represented as >10 h in the plot. Data are represented as mean ± SEM.

The Aβ_40_/Aβ_42_ amyloid formation from monomeric peptides into insoluble amyloid fibrils in the absence or presence of NCAM-PrP peptides was monitored by the ThT kinetics assay (Figure 4). ThT is a small molecule that recognizes amyloid structures with subsequent changed fluorescence properties which are easily detected as an increase in fluorescence intensity over time. The final ThT fluorescence intensity of the sample is often referenced to correspond to the amount of amyloid material.

The Aβ_42_ monomeric peptides (5 μM) aggregated into amyloid fibrils with a typical sigmoidal kinetic curve with an aggregation half-time (τ_½_) of about 4 h (black curves). In the presence of 5 μM NCAM-PrP peptide, the τ_½_ was increased to approximately 7 h (dark blue curves), as shown in Figure 4A. The final ThT intensity level was also reduced for Aβ_42_ peptides incubated in the presence of NCAM-PrP, which may suggest a reduction of ThT-active aggregates present. This observation was confirmed by atomic force microscopy (AFM) imaging experiments of the samples after the ThT kinetic experiment, where the Aβ_42_ sample shows amyloid fibrils with a diameter of approximately 10 nm whereas the Aβ_42_ + NCAM-PrP samples do not contain any typical amyloid fibrils, rather amorphous aggregates (Figure 4B).

Aβ_42_ peptides, alone or with NCAM-PrP, were also incubated over time with 150 mM NaF to study the influence of ionic strength. The rate of amyloid aggregation of Aβ_42_ peptides in the absence of NCAM-PrP increased with increasing ionic strength, with a τ_½_ of approximately 2.5 h (gray curves), in line with previous observations (Abelein et al., 2016; Meisl et al., 2017). In the presence of NCAM-PrP, the kinetic curves did not show any measurable increase in ThT fluorescence intensity (light blue curves). A decrease in ThT activity may originate from decreased amounts of amyloid material, or from changed ThT properties upon the experimental conditions. The impact of ionic strength was verified by several independent measurements, and with 80 mM sodium phosphate buffer instead of added NaF salt (data not shown). The effect of NCAM-PrP on the amyloid aggregation of 12 μM Aβ_40_ showed a similar trend as observed for Aβ_42_, namely increasing τ_½_ values in the presence of NCAM-PrP. The τ_½_ increased to approximately 8 h (dark green curves) in the presence of an equimolar concentration of NCAM-PrP, compared to approximately 6 h for Aβ_40_ alone (black curves). The τ_½_ decreased to 2 h in the presence of 150 mM NaF (gray curves) without NCAM-PrP. However, the increase in ionic strength did not significantly influence the attenuating effect of the NCAM-PrP (light green curves). This observation most likely excludes electrostatic interactions as the major type of interaction between Aβ and NCAM-PrP.

To shed more light on the mechanistic details of the NCAM-PrP effect on Aβ amyloid formation, we performed kinetic experiments under seeded conditions. Seeding experiments are used to distinguish whether primary or secondary nucleation processes during Aβ amyloid formation are most affected by the studied modulator. In the presence of seeds, there are many sites of fibril ends and surfaces provided. In this situation the elongation and secondary nucleation are dominant, whereas the contribution of primary nucleation is minimal. In Figure 4D 5 μM monomeric Aβ_42_ peptides aggregated into amyloid fibrils as expected. In the presence of 10% pre-formed seeds, the amyloid formation was enhanced and reached the plateau phase before the samples in the absence of seeds left the lag phase, indicative of dominating secondary nucleation processes in line with previous studies (Cohen et al., 2012). In contrast, the effect of pre-formed seeds was abolished in the presence of NCAM-PrP. This is an intriguing observation, indicating that the Aβ secondary nucleation processes are suppressed by NCAM-PrP.

### Atomic and molecular interactions between NCAM-PrP and Aβ

#### NMR spectroscopy

In water at pH 5.4, the NCAM-PrP is largely unstructured with a dispersed 1D NMR spectrum (Figure S6). However, in a buffered solution at physiological pH, the signals in the 1D NMR spectrum of NCAM-PrP peptide are significantly reduced and broadened, indicative of the formation of larger peptide structures. An instant precipitation of the peptide in the sample tube was also observed. These larger peptide structures, or aggregates, are subject to further investigation. However, hydrogen exchange effects contributing to the observations may not be rejected.

**Figure 5:**
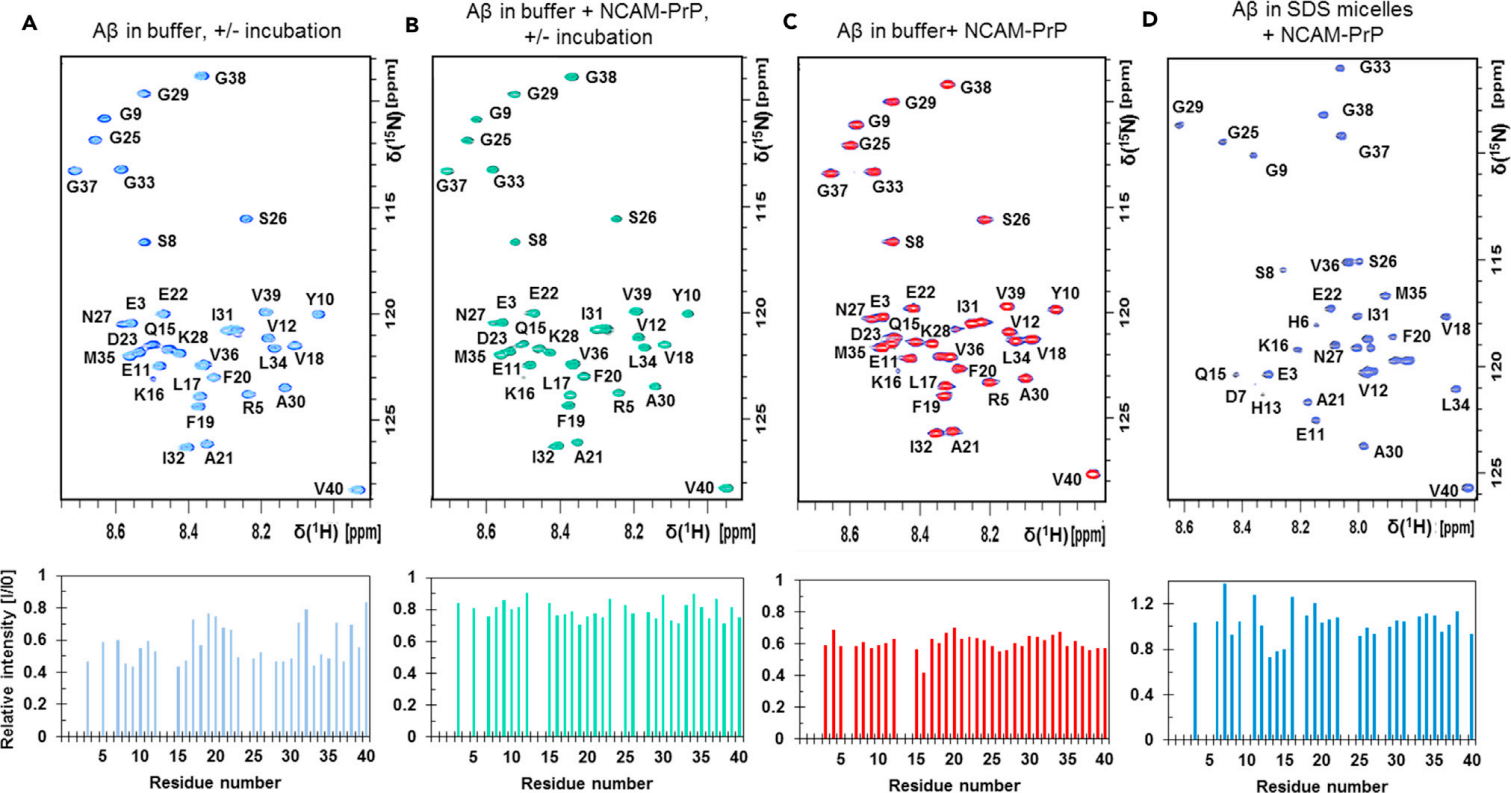
The monomeric NCAM-PrP interaction with monomeric Aβ_40_ peptides was studied using NMR spectroscopy (A and B) In order to study the monomeric NCAM-PrP interaction with monomeric Aβ_40_ peptides over time, samples of 20 μM ^15^N-Aβ_40_ peptides in 20 mM MOPS buffer pH 7.35 were incubated for 48 h at +37°C and shaking conditions (200 rpm) in the (A) absence and (B) presence of 5 μM NCAM-PrP. ^1^H-^15^N-HSQC spectra were recorded at +5°C before and after the incubation, where only the monomeric ^15^N-labelled Aβ_40_ peptides are visible. In (A) and (B) are the spectra before/after incubation shown, together with relative intensity plots comparing the intensity of the amplitude height of the amide crosspeaks after/before the incubation. (C) ^1^H-^15^N-HSQC spectra of 70 μM monomeric ^15^N-Aβ_40_ peptides in the absence and presence of 30 μM NCAM-PrP in 50 mM sodium phosphate (NaP) buffer pH 7.3 were recorded and presented as overlayed spectra and with a relative intensity plot. (D) The interaction between monomeric Aβ peptides and NCAM-PrP was further studied in the presence of SDS micelles. 84 μM ^15^N-Aβ_40_ peptides and 50 mM SDS-d25 micelles in 20 mM NaP buffer pH 7.3 was used, and ^1^H-^15^N-HSQC spectra were recorded at +25°C. 84 μM monomeric NCAM-PrP peptides were titrated onto the ^15^N-Aβ_40_ sample. See also Figures S4 and S6–S8.

2D NMR ^1^H-^15^N-HSQC experiments were recorded to study the concentration-dependence of the interaction between Aβ and NCAM-PrP at residue-specific resolution (Figure 5). Monomeric NCAM-PrP was titrated onto a ^15^N-Aβ_40_ peptide sample (one titration step presented in Figure 5C) with subsequent loss of signal of the amide crosspeaks generally distributed over the peptide sequence. A global fit analysis of the corresponding intensity loss during the titration, with all residues included, was performed (Figure S7). An overall dissociation constant (Tiiman et al., 2016) of approximately 160 ± 120 μM was determined. However, the reason for the loss of signal due to increasing concentrations of NCAM-PrP is not fully clear. We performed CPMG relaxation dispersion experiments (Carr and Purcell 1954; Meiboom and Gill 1958; Tollinger et al., 2001; Wallin et al., 2020) to be able to detect chemical exchange effects between Aβ and Aβ:NCAM-PrP complexes (Figure S8). No exchange was detected in the relaxation dispersion profiles. These observations suggest that the loss of signal is not due to chemical exchange on the milli-to microsecond timescale. There may be several explanations for this observation: (1) The interactions with monomeric Aβ peptides are not strong enough; (2) the interaction between Aβ and NCAM-PrP may be on a higher level of Aβ aggregates instead of monomeric Aβ; or (3) the interaction between monomeric Aβ and monomeric NCAM-PrP does not occur on the NMR timescale observable with the relaxation dispersion experiments using CPMG-based pulse sequences (milli- to microsecond timescale) that was used here. It is worth noting that a low concentration of NCAM-PrP was used since high NCAM-PrP concentrations in the sample induced precipitation.

From the NMR experiments an instant effect on Aβ from the interaction between Aβ and NCAM-PrP was clearly observed. To study the effect over time samples of Aβ alone and in the presence of NCAM-PrP were incubated for two days. HSQC spectra were recorded before and after the incubation. As expected, the sample with Aβ alone started to aggregate during the incubation, which was characterized by an overall signal intensity decrease (Figure 5A). However, the sample with both Aβ and NCAM-PrP did not aggregate (observed as loss of signal intensity) to the same degree as without NCAM-PrP (Figure 5B). Importantly, even if the signal intensity remained higher over time for Aβ in the presence of NCAM-PrP, an immediate loss of Aβ signal after titration with NCAM-PrP at time zero was observed.

Sodium dodecyl sulfate (SDS) micelles are often used as a simple membrane-mimetic to constrain the Aβ peptide in a monomeric conformation and prevents its aggregation ([SDS micelles]>[Aβ]). Both Aβ and NCAM-PrP peptides adopt α-helical secondary structures in such membrane-mimicking environments (Figure S4) (Henning-Knechtel et al., 2020). Here we used ^1^H-^15^N-HSQC experiments to investigate if Aβ and NCAM-PrP interact in SDS micelles. 50 mM SDS was added to a sample of ^15^N-Aβ peptides and NCAM-PrP was titrated onto this sample (Figure 5D). Only minor overall changes were observed, with a more pronounced effect for residues 13-15 as signal intensity attenuation at a 1:1 Aβ:NCAM-PrP ratio was detected.

#### Aβ and NCAM-PrP form large co-clusters and few heterooligomers in aqueous solution

The NCAM-PrP peptide exhibited a highly charged and wide charge state distribution in native electrospray mass spectrometry analysis (Figure S9), indicative of a very flexible and unfolded peptide ensemble. The high charge states are also explained by the many basic sites in the peptide sequence (solution state net charge of +6 at pH 7). NCAM-PrP is observed as a predominantly monomeric peptide, with only a small fraction of dimeric species observed under low ionic strength. Larger unresolved structures were also observed under low ionic strength, which could be dissociated into monomeric NCAM-PrP by collisional activation in MS/MS (Figure S9).

**Figure 6:**
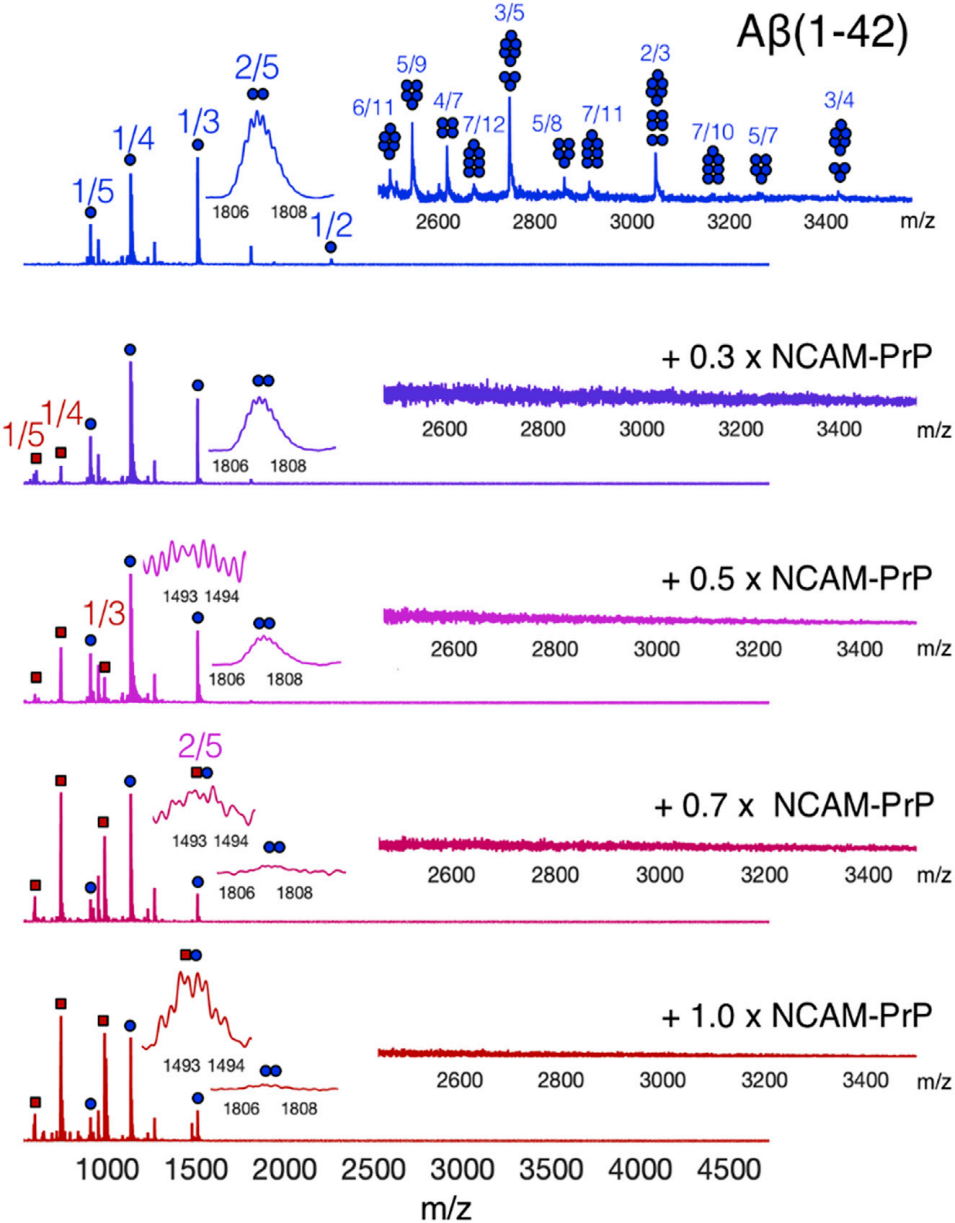
Mass spectra of 14 μM Aβ_42_ in 20 mM ammonium acetate pH 8.3 with varying amounts of NCAM-PrP Blue circles indicate Aβ signals and red squares indicate NCAM-PrP signals. Low intensity oligomeric signals are magnified in the inserts. See also Figures S9 and S10.

Aβ_42_ is observed in native mass spectrometry as a mostly monomeric peptide with a rich population of small and heterogeneous oligomers (Figure 6), in agreement with previous reports (Bernstein et al., 2009; Pujol-Pina et al., 2015). The low intensity of the observed oligomers is in agreement with findings that the oligomer population does not reach more than a few percent of the total Aβ peptide population in a simple aqueous *in vitro* solution (Michaels et al., 2020). The Aβ_42_ oligomer signals quickly disappear upon titration with NCAM-PrP (Figure 6). The titration predominantly affects larger Aβ oligomers, with all signals associated with these species disappearing even after addition of only 0.3 molar equivalents of NCAM-PrP to the Aβ_42_ sample. The +5 charged Aβ_42_ dimer signal is more resilient to the titration and can be detected at low intensity even after addition of 1 molar equivalent of NCAM-PrP.

Monomeric NCAM-PrP signals increase in intensity upon increasing the concentration of that peptide under constant Aβ_42_ concentration. The charge state distribution of the Aβ_42_ monomer shift slightly to higher charge states upon addition of NCAM-PrP, with the most intense charge state shifting from +3 to +4. This could indicate a slight shift in the solution state ensemble, as electrospray charging is proportional to the solvent accessible surface area of the peptide. Heterooligomer formation between the two peptides is not seen to any greater extent, only a low intensity dimeric heterooligomer is observed after addition of 0.7 or more molar equivalents of NCAM-PrP. The nanospray emitters used to introduce the samples into the mass spectrometer became increasingly prone to clogging upon addition of higher amounts of NCAM-PrP to the Aβ_42_ sample, indicating the formation of larger aggregates. The interaction between the two peptides in an aqueous *in vitro* solution might therefore take place at size scales not detectable by mass spectrometry.

#### Aβ and NCAM-PrP form heterooligomers in a membrane-mimicking environment

Signal peptides are membrane-embedded *in vivo* and it is therefore possible that the interactions between NCAM-PrP and Aβ take place in the membrane under cellular conditions. Interactions between Aβ and cellular membranes have been correlated with toxicity, motivating further studies of Aβ in membrane environments. Co-incubation with zwitterionic micelles has previously been demonstrated to increase the population of Aβ_42_ oligomers (Österlund et al., 2019), and such micelles can be considered as a simple membrane-mimicking environment (Österlund et al., 2019). As both Aβ and NCAM-PrP are membrane-interacting peptides, their oligomerization in membrane-mimicking micelles was here studied in a similar way using native mass spectrometry. The micelles were then stripped away inside the mass spectrometer using collision-induced dissociation, revealing the mass of oligomers without bound detergent. The zwitterionic LDAO detergent was added to each peptide at 2 x critical micelle concentration (2 x 2 mM), and to an equimolar mixture of the two peptides.

**Figure 7:**
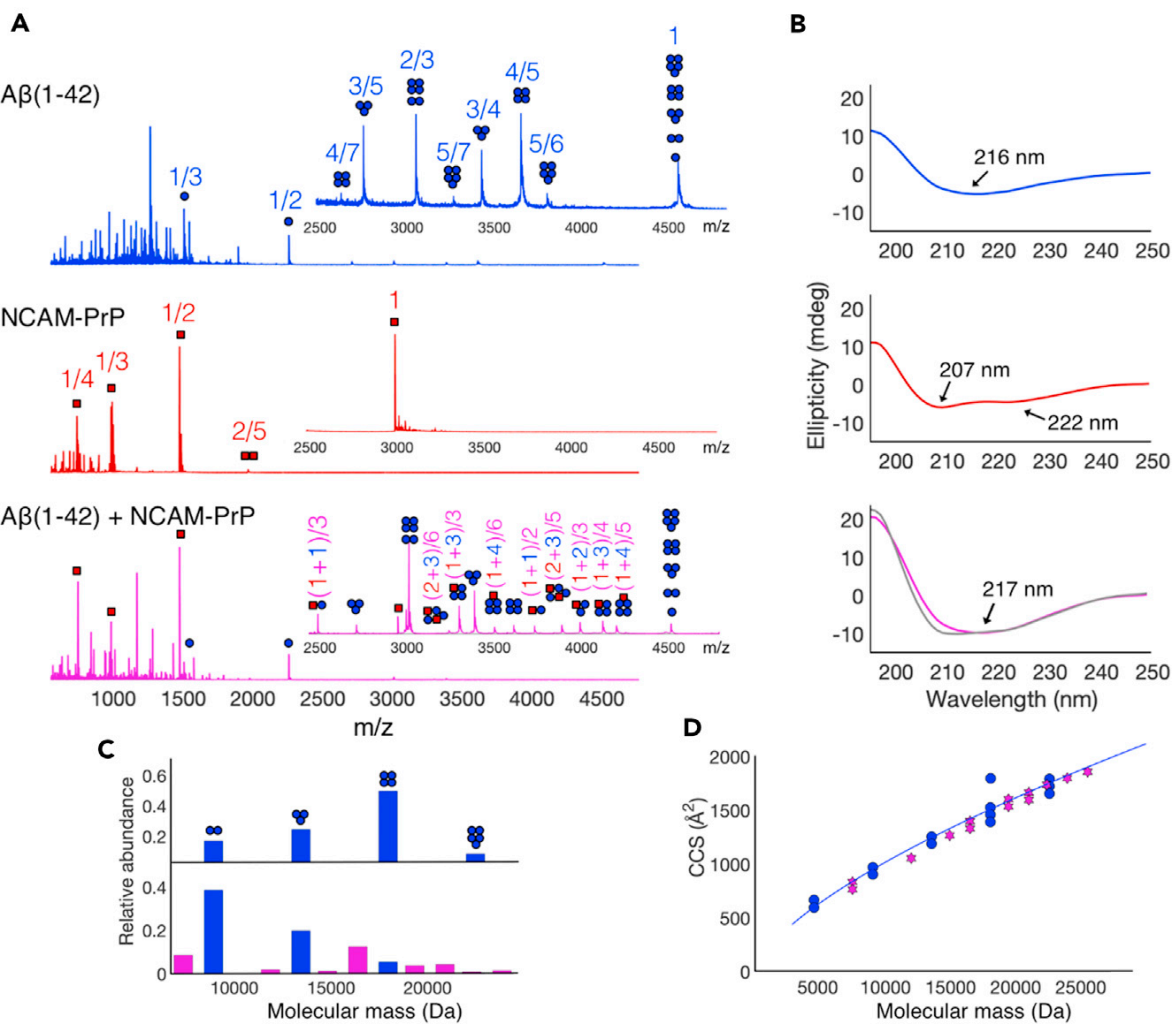
Mass spectrometry and circular dichroism spectroscopy of Aβ and NCAM-PrP in a micellar environment (A) Mass spectra after CID activation of 14 μM Aβ_42_, 14 μM NCAM-PrP and 14 μM Aβ_42_ + 14 μM NCAM-PrP after co-incubation with 4 mM LDAO micelles (2 x CMC). Blue circles indicate Aβ signals and red squares indicate NCAM-PrP signals. Numbers indicate the oligomer to charge ratio (n/z), heterooligomers are annotated as (n_NCAM-PrP_ + n _Aβ_)/z. (B) CD data of 14 μM Aβ, 14 μM NCAM-PrP and 14 μM Aβ/NCAM-PrP (1:1 ratio) in LDAO micelles. The gray spectrum in the bottom panel originates from adding the spectra of Aβ and NCAM-PrP theoretically together by their individual spectra above. (C) Relative abundance of each oligomeric species for Aβ_42_ (top panel) and Aβ/NCAM-PrP (bottom panel). Blue bars show Aβ homooligomers, purple bars show heterooligomers. (D) Collision cross section (CCS) as a function of molecular mass for Aβ homooligomers (blue) and heterooligomers (purple). The fitted blue line (y = Ax^2/3^) for Aβ homooligomers and illustrates the spatially isotropic growth of both homo- and heterooligomers. See also Figure S11.

Such co-incubation with micelles resulted in an increase in Aβ_42_ oligomer signals (Figure 7A, top panel), in agreement with previous findings (Österlund et al., 2019). Under these conditions, Aβ oligomers between dimers and pentamers were clearly observed, with the tetramer being the most populated oligomeric state (about 50% of the oligomeric population). The Aβ_42_ sample in LDAO micelles exhibits mainly β-sheet structures in a CD spectrum (Figure 7B top panel), in agreement with previous results that demonstrated formation of so-called β-sheet pore-forming oligomers upon co-incubation in zwitterionic micelles (Österlund et al., 2019). NCAM-PrP, on the other hand, exhibits α-helical secondary structures in the same micelle environment (Figure 7B, middle panel), typical of monomeric peptides in micelles. Mass spectrometry confirms that NCAM-PrP remains mostly monomeric in this membrane environment, with a small amount of dimers also detected. The equimolar mixture of Aβ_42_ and NCAM-PrP, however, displayed a rich population of both Aβ_42_ homooligomers and Aβ:NCAM-PrP heterooligomers. This is in stark contrast to the observations made in a simple aqueous solution (Figure 6). These heterooligomers were assigned based on the theoretical m/z calculated for different heterooligomers, and the composition was confirmed by tandem MS analysis. An example of MS/MS analysis of the (1 + 3) oligomer signals are shown in the supporting information (Figure S10). We have previously demonstrated that the oligomerization of Aβ_42_ in micelles is sequence specific (Österlund et al., 2019). Here we also show that the insertion and co-oligomerization with NCAM-PrP is possible for Aβ_42_, whereas a scrambled version of Aβ_42_ (Aβ_42_^Scr^) peptide does not form heterooligomers with NCAM-PrP in LDAO micelles (Figure S11).

All detected heterooligomers were Aβ-rich, with a composition of 50-80% Aβ_42_, meaning that only one or two NCAM-PrP peptides attach to each Aβ oligomer. This is in agreement with the observed higher oligomerization propensity of Aβ compared to NCAM-PrP. This indicates that mostly monomeric NCAM-PrP peptides attach to Aβ oligomers, which shifts the distribution of Aβ oligomers toward smaller assemblies. The CD spectrum of the Aβ:NCAM-PrP sample in LDAO micelles clearly exhibits large amounts of β-sheet structures (Figure 7B lower panel, purple trace). The theoretical spectrum for the mixture contains higher amounts of helical structure (Figure 7B, lower panel, gray trace) indicated by a minimum at lower wavelengths. This means that some peptides are converted from helix to β-sheet upon mixing of the two peptides. It can also be noted that the intensities of the CD spectrum of the peptide mixture are higher in the micelle environment compared to in a simple aqueous solution (Figures 2D and 2E).

The binding of NCAM-PrP to Aβ changes the mass distribution of the oligomer population in the micelles, shifting the most populated Aβ homooligomer from the tetramer to the dimer (Figure 7C). High-mass heterooligomers are instead formed, which are however of lower relative abundance compared to the abundance of the Aβ homotetramer in absence of NCAM-PrP. The largest observed heterooligomer is the heterohexamer of 4 Aβ_42_ and 2 NCAM-PrP (24 kDa), carrying 6 charges (n/z = (1 + 2)/3). A shape factor known as the collision cross section (CCS), providing structural information and physical size of the complexes, can be determined from ion mobility data. CCS values showed that heterooligomers fall on the same isotropic growth curve (CCS = x^2/3^) as Aβ homooligomers. This indicates that the size of heterooligomers grows as spheres, and that their shape is approximately the same as the shape of an Aβ homooligomer of a similar size.

## Discussion

Aggregation of Aβ peptides is associated with Alzheimer’s disease and modulation of such processes is of high interest for future therapeutics. Protein-protein interactions-based therapeutics are currently recognized for their potential usage both in practice and as promising agents in clinical trials (Dimitrov 2012). For AD treatment passive immunization strategies were recently initiated in terms of drug candidates targeting Aβ peptides, with a few promising antibodies presented (Panza et al., 2016; Sevigny et al., 2016; Logovinsky et al., 2016; Selkoe and Hardy 2016). Despite achievements with respect to desirable clinical endpoints, the bioavailability of the antibodies is low and high doses are required. CPPs have the advantage of high bioavailability, high target specificity, and selectivity, coupled with the ability to readily enter cells whether alone or coupled with a therapeutic cargo (Guidotti et al., 2017; Raucher and Ryu 2015; Ramsey and Flynn 2015). CPPs have been used for efficient delivery of amyloid inhibitors (Kokotidou et al., 2019). The NCAM-PrP peptide construct used in this study has CPP-properties (Henning-Knechtel et al., 2020; Magzoub 2020). One complementary and intriguing property of the NCAM-PrP peptide is therefore that it has both CPP and anti-amyloid properties. Its presence in a cell membrane may well be crucial for its effect on the Aβ-induced neurotoxicity (Kotler et al., 2014; Österlund et al., 2019; Butterfield and Lashuel 2010; Henning-Knechtel et al., 2020).

**Figure 8:**
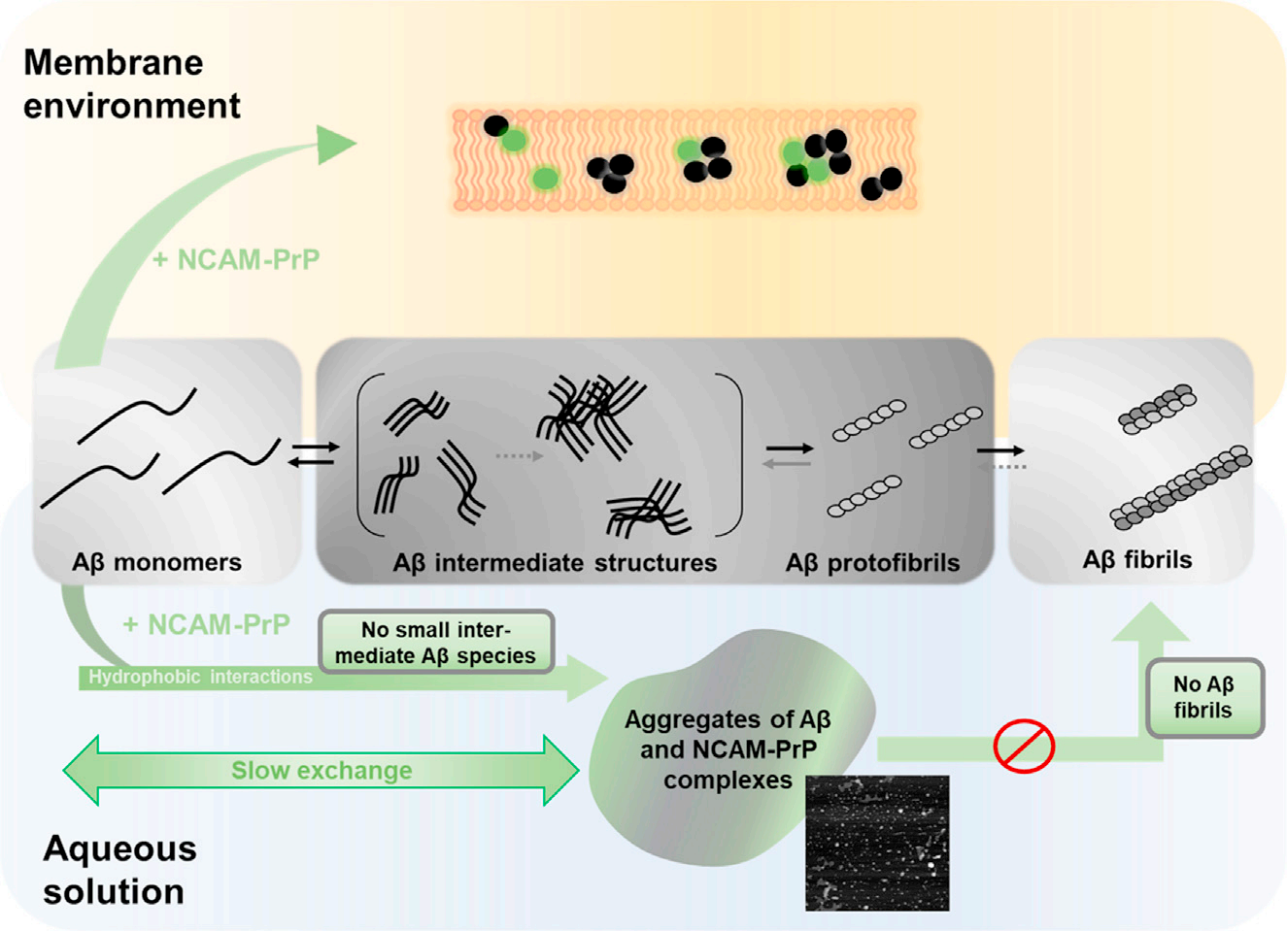
Schematic illustration of the NCAM-PrP inhibitory effect of Aβ peptide amyloid fibrillization The fibrillization pathway of Aβ is exemplified in the middle of the figure in black (Aβ) and gray colors. The NCAM-PrP effect is showed in green color, in a membrane-mimetic environment (yellow) above and in aqueous solution (blue) below the Aβ peptide fibrillization pathway, respectively. In aqueous solution, the interaction between Aβ monomers and NCAM-PrP monomers drastically results in formation of large aggregates not visible in solution NMR spectra or native mass spectrometry. Hydrophobic interactions are important for the formation of these large complexes. The Aβ:NCAM-PrP complexes are visualized as “blobs” in AFM images and are not building blocks for further amyloid fibrillization processes. In a zwitterionic membrane-mimetic environment a different behavior is observed, probably due to the hydrophobic nature of the lipids. A sample of Aβ and NCAM-PrP in a membrane both forms Aβ:NCAM-PrP heterooligomers and Aβ homooligomers.

This is an *in vitro* study of the physio-chemical nature of the Aβ:NCAM-PrP interaction. Circular dichroism-, NMR- and fluorescence spectroscopy, as well as mass spectrometry, were used to gain in-depth molecular insights of the NCAM-PrP amyloid inhibition (Figure 8). Aβ and NCAM-PrP clearly interact based on our biophysical findings. The overall NCAM-PrP inhibitory effect on Aβ amyloid formation occurs on several structural levels ranging from a monomer-monomer interaction to interactions including larger Aβ aggregates. Interestingly, the Aβ-NCAM-PrP interaction is affected by the presence of a membrane-mimetic environment.

### The Aβ:NCAM-PrP interaction in aqueous solution

The Aβ amyloid aggregation behavior is changed in the presence of the NCAM-PrP peptide by promoting formation of aggregates inert for further amyloid formation. NCAM-PrP hinders Aβ’s conversion into β-sheet typical for Aβ fibrillization simultaneously as the typical random coil structures of monomeric Aβ disappears (Figure 2D). The Aβ:NCAM-PrP co-aggregate does not, however, retain the random coil conformation of monomeric Aβ at equimolar concentrations. This inhibition of amyloid formation is similar regardless of the Aβ isoform (Figure 2 and Figure S5), and the absence of amyloid structures has been confirmed using ThT kinetics assays, AFM (Figure 4), FTIR measurements (Figure 3), and TEM (Henning-Knechtel et al., 2020). The Aβ:NCAM-PrP co-aggregate could be dissolved by adding SDS detergent (Figure 4C), indicating that they are less ordered and stable than pure Aβ aggregates which become increasingly SDS-resistant during aggregation (Coalier et al., 2013). Such a loose aggregate might therefore be easier for cellular degradation systems to disaggregate compared with mature amyloid structures.

Electrostatic and hydrophobic interactions are important for Aβ peptide aggregation. At physiological pH, the net charge of the Aβ peptide is about −2.7, whereas the NCAM-PrP peptide is relatively highly positively charged (+6). Attenuation of Aβ amyloid formation by interactions with positively charged proteins and molecules are common (Assarsson et al., 2014; Wallin et al., 2018, Wallin et al., 2020; Österlund et al., 2018). The NCAM-PrP inhibitory effect on Aβ fibrillization was neither affected by an increase in ionic strength nor in the presence of seeds (Figure 4). The major binding contribution for the Aβ:NCAM-PrP complex is hence not likely solely due to electrostatic interactions (Figures 4A and 4C), but with hydrophobic interactions possibly at least equally important. On the other hand, the positively charged hexapeptide mPrP_23-28_ at the C-terminal part of NCAM-PrP cannot be removed without any loss of the amyloid inhibitory effects (Henning-Knechtel et al., 2020). It should, however, be considered that interactions with intracellular Aβ could take place after signal peptidase processing of the construct. This means that electrostatically driven interactions with the hexapeptide could be more pronounced *in vivo*, after removal of the hydrophobic signal sequence which contributes to interactions in our *in vitro* experiment performed on the intact construct.

The absence of an increased amyloid formation rate in the presence of pre-formed seeds indicates a suppression of secondary nucleation processes. In general, inhibition of secondary nucleation processes may be achieved by reducing the available fibril surface necessary for catalysis of new aggregates (Meisl et al., 2014; Cohen et al., 2013). The strong inhibitory effect of NCAM-PrP may, therefore, be the result of suppressed secondary nucleation processes via formation of Aβ:NCAM-PrP intermediate complexes (Figures 2, 4, and 5). Such inhibition of secondary nucleation is linked to a decrease in toxicity, as the amount of toxic prefibrillar aggregates is greatly decreased (Flagmeier et al., 2020). The inhibition of Aβ aggregation in the presence of NCAM-PrP is similar to the impact of chaperone proteins on Aβ aggregation (Cohen et al., 2015; Leppert et al., 2019; Chen et al., 2017; Scheidt et al., 2019; Wang et al., 2020). The chaperone-functional BRICHOS domain, consisting of about 100 amino acids, is found in several proteins related to amyloid formation and is an example of a chaperone which inhibits secondary nucleation (Leppert et al., 2019; Chen et al., 2017). Seeded Aβ aggregation performed in the presence of BRICHOS looks similar to seeded Aβ aggregation in the presence of NCAM-PrP (Figure 4). BRICHOS forms a coating along the Aβ fibril surface, which has been verified with imaging techniques (Cohen et al., 2015). In addition, the NCAM-PrP effect on Aβ aggregation is similar to the effects of full-length native Tau protein (Wallin et al., 2018). Like NCAM-PrP, Tau protein is unstructured and positively charged at physiological pH.

The native MS experiments did not detect a substantial amount of Aβ:NCAM-PrP heterooligomers (Figure 6). However, formation of larger Aβ:NCAM-PrP complexes not observed in these measurements cannot be excluded, as is also evident by precipitation upon mixing at high NCAM-PrP concentrations. The NMR measurements illustrate how monomeric Aβ peptides interact with the NCAM-PrP peptide since mainly the monomeric Aβ signals are visible in the spectra. NCAM-PrP instantly reduces the Aβ monomeric signals uniformly with a weak monomer-monomer or monomer-co-aggregates interaction without chemical shift changes, and over time the monomeric Aβ signal left is preserved in the presence of NCAM-PrP without further aggregation (Figure 5). The preference of NCAM-PrP for certain Aβ residues important for the interaction is still broad and is not distinguishable (Figure 5). Hence, the Aβ:NCAM-PrP interaction reduces the monomeric Aβ concentration by forming complexes that do not promote further amyloid aggregation of monomeric species left in the sample. Notably, as the NCAM-PrP concentration increases during titration onto an Aβ sample, the monomeric NCAM-PrP signal intensities also increase in the MS spectra. This observation may indicate an exchange of peptide interactions rather than an instant precipitation out of the solution for both peptides. The monomer-monomer interaction (or Aβ monomer-aggregated complex) is not on the micro-millisecond timescale (Figure S8) and is likely not exhibiting fast exchange due to the lack of observable chemical shift changes in the HSQC spectra. We suggest that slow dynamic exchange is present for the Aβ-NCAM-PrP complex, which is supported by the inhibition of amyloid formation without complete inhibition.

Taken together, these results indicate NCAM-PrP-induced formation of large co-aggregates with Aβ, via mostly hydrophobic interactions. Such co-aggregates are not amyloid fibrillization−competent and are dissolved by SDS treatment.

### The Aβ:NCAM-PrP interaction in a membrane environment

The membrane perturbational effects of the NCAM-PrP peptide are dependent on the lipid headgroup charge used in the LUV model system (Figure 1). The highest perturbational effect, observed as increased leakage in the presence of NCAM-PrP, was achieved for zwitterionic LUVs with 100% POPC (Figure 1). In contrast, highly negatively charged LUVs were not disrupted by the NCAM-PrP peptide. The Aβ:NCAM-PrP complex was able to reduce the NCAM-PrP leakage efficiency in both 100% POPC LUVs and in moderate negatively charged LUVs (Figure 1). The mechanism by which the NCAM-PrP peptide perturbs lipid membranes is still not fully understood. Several routes/pathways may be present, such as transient pore formation and endocytosis. The antibacterial properties of the NCAM-PrP peptide have not been investigated. Our results show more leakage for zwitterionic lipid membranes, such as for eukaryotic plasma membranes, compared with negatively charged membranes often found in prokaryotes. There are amphiphilic peptides with membrane perturbational effects like the antimicrobial peptide (AMP) LL-37 with a clear preference for negatively charged membranes (Zhang et al., 2010). The NCAM-PrP ability to induce leakage is similar to the leakage efficiency of the hydrophobic Tp10 peptides, analogs to transportan, with higher leakage in zwitterionic LUVs than in negatively charged LUVs (Bárány-Wallje et al., 2007). Membrane-leakage can occur through different mechanisms (Matsuzaki 2019). No NCAM-PrP oligomer larger than the dimer is observed here for this peptide alone, either in solution or in the membrane environment (Figures S9 and 7). This is in contrast to the Aβ peptide where many oligomeric states are detected in membrane-like environments (Figure 7). Such membrane-bound oligomers are assumed to form pore-like structures through which leakage can occur (Sciacca et al., 2012; Österlund et al., 2019; Quist et al., 2005; Ciudad et al., 2020), but under our experimental conditions only minor leakage from Aβ alone was detected, compared to the NCAM-PrP peptide in LUVs (Figure 1B). It is worth notice that calcein leakage has been found to only report on Aβ mediated disruption of LUVs by large aggregates, and not by formation of small oligomeric pores such as those detected by MS (Sciacca et al., 2012). The structural states of Aβ are probably of high importance for the membrane interaction and/or perturbation, as well as membrane composition, both *in vitro* and *in vivo*. Usage of different techniques have all reported very minor to significant leakage by Aβ_40_ and Aβ_42_ (Sciacca et al., 2012; Bode et al., 2017; Qiang and Doherty 2018). Our results clearly indicate an interaction between Aβ_42_ and NCAM-PrP in the presence of a membrane. In addition, direct Aβ_42_ membrane perturbational effects detected as calcein leakage may not be the main route of adverse Aβ consequences, and other cellular effects may also be present.

The Aβ:NCAM-PrP interaction in the presence of membrane-mimetic environments was further studied. Both Aβ and NCAM-PrP peptides instantly adapt to α-helical secondary structures independently of each other in negatively surface charged SDS micelles (Figure S4). Together in SDS micelles, only a minor Aβ:NCAM-PrP interaction was observed (Figure 5). In contrast, usage of zwitterionic LDAO detergent micelles in the Aβ samples supplemented with NCAM-PrP generated both Aβ homooligomers and Aβ:NCAM-PrP heterooligomers (Figure 7). The observed discrepancies for the interaction in different membrane environments, i.e., SDS micelles, LDAO detergent micelles, and in LUVs, may originate from the different properties of the used membrane models. There are differences in net charge as well as differences in diameter and surface properties/curvatures, all important for membrane-peptide interactions and subsequently the peptide-peptide interaction studied here. In negatively charged membranes, the highly positively charged NCAM-PrP peptide may have higher preference for membrane surface interactions rather than Aβ peptide-peptide interactions in the hydrophobic part of the membrane. Such surface interactions can explain the lower leakage efficiency detected in negatively charged LUVs (Figure 1). Co-aggregation of Aβ and NCAM-PrP seems to occur on a different level in a membrane environment compared to in an aqueous solution. Formation of large aggregates is observed in aqueous solution, which precipitate out of the solution. Such large precipitates are unlikely to be the reason why Aβ mediated toxicity would decrease in living cells upon addition of NCAM-PrP peptides. The cell contains many possible interaction partners that could be important for the observed *in vivo* effects. The introduction of a hydrophobic membrane environment seems to modulate the formation of aggregates into smaller and more specific co-clusters as hydrophobic peptide-membrane interactions compete with hydrophobic peptide-peptide interactions in solution. This is observed both as a higher abundance of small heterooligomers and higher intensities in CD spectra (Figure 7). This observation indicates a different behavior in aqueous solution versus in a zwitterionic membrane mimetic environment. Because endogenous signal peptides are inserted as transmembrane helices upon translation, it is highly likely that the hydrophobic NCAM_1-19_ segment would partition into the membrane also upon exogenous addition to cells. This means that the peptide effectively acts as a CPP. It has previously been reported that the KKRPKP hexapeptide motif in the prion protein readily interacts with the Aβ peptides (Younan et al., 2013; Chen et al., 2010), and details about this interaction are still subject for further investigation. Since the inhibitory effect of NCAM-PrP occurs both extra- and intracellularly in cell studies (Henning-Knechtel et al., 2020; Söderberg et al., 2014), our observations of the Aβ-NCAM-PrP interaction in the presence of membrane mimetics are highly relevant to the NCAM-PrP effects during biological/physiological conditions. It is possible that the observed anti-amyloid effect of the construct *in vivo* is due to interactions extracellularly, intracellularly, and/or in the membrane.

### Concluding remarks

The effect of the NCAM-PrP peptide on several amyloidogenic systems, such as the Aβ peptide, the prion protein (Söderberg et al., 2014), and the S100A9 protein (Pansieri et al., 2019), is intriguing. The NCAM-PrP peptide is not toxic at the concentrations studied, and NCAM-PrP attenuates Aβ-induced cytotoxicity in cells (Henning-Knechtel et al., 2020). Similar anti-amyloid effects have been seen also when the PrP hexapeptide is replaced by the KKLVFF sequence inspired by the Aβ peptide (Henning-Knechtel et al., 2020; Pansieri et al., 2019; Gielnik et al., 2021). Here, we present further molecular insights into the Aβ:NCAM-PrP interaction responsible for inhibition of amyloid formation. Our data suggests that NCAM-PrP shifts the equilibrium of Aβ aggregation competent units toward species not competent for further amyloid aggregation. Interestingly, NCAM-PrP increases the amyloid aggregation rate of S100A9 (Pansieri et al., 2019), a folded protein in its native state, which is in stark contrast with the construct’s effects on the intrinsically disordered Aβ peptide or prion protein. A possible explanation for this is that NCAM-PrP interacts specifically with amyloidogenic sequences, which are normally buried in native protein structures. This suggests that NCAM-PrP unfolds/dissolves highly structured protein structures, in addition to promoting species not leading to further amyloid aggregation. Another question to address in future studies is to elucidate the critical component in the NCAM-PrP peptide sequence that exhibits this strong amyloid inhibitory effect in several amyloidogenic systems in solution, in membrane-mimicking environments, and in cells. It has been proposed that an important amyloid inhibiting interaction originates from the “FF”-motif (Mason and Buell 2019; Jarmuła et al., 2021), both in the Aβ amino acid sequence and of another modulator of Aβ fibrillization, namely LL-37 (De Lorenzi et al., 2017). Interestingly, an “FF”-motif is also present in the NCAM-PrP construct peptide, in the signal NCAM_1-19_ sequence. The importance of this motif for the Aβ:NCAM-PrP interactions remains to be determined. The cellular fate of the peptide also warrants further investigation, to determine if the anti-amyloid effect is attributed to the intact or cleaved form of the peptide. Nevertheless, the strong amyloid inhibitory effect is accompanied by CPP activity, providing this peptide construct with properties for potential future therapeutic possibilities. The Aβ:NCAM-PrP interaction affects the amyloid aggregation and this interaction also occurs in a membrane-mimetic environment.

### Limitations of the study

One potential strategy to combat Alzheimer’s disease is to target Aβ peptide misfolding and aggregation with peptide-peptide interactions. Here, the NCAM-PrP peptide is studied, a peptide with amyloid-inhibiting effects on several amyloidogenic systems. This is an *in vitro* study using model systems to investigate the molecular processes of biologically relevant amyloid formation implicated in disease, both in aqueous solution and in membrane-mimetic models. The simple model systems *in vitro* are used as a model, with limitations due to the simplicity to explain complex chemical and biological processes.

## STAR★Methods

### Key resources table

**Table:** 

REAGENT or RESOURCE	SOURCE	IDENTIFIER
**Chemicals, peptides, and recombinant proteins**		

Recombinant Amyloid-β_1-42_ (Aβ_42_) wild type peptide	rPeptide, Watkinsville, USA	A-1163-2
Recombinant Amyloid-β_1-40_ (Aβ_40_) wild type peptide	AlexoTech AB, Umeå, Sweden	AB-100-10
Uniformly isotopically ^15^N -labelled Amyloid-β_1-40_ (Aβ_40_)	AlexoTech AB, Umeå, Sweden	AB-100-05
Synthetic NCAM1_1-19_-mPrP_23-28_ (NCAM-PrP) peptide construct	PolyPeptide Group, Strasbourg, France	SP120001B
1-palmitoyl-2-oleoyl-glycero-3-phosphocholine; POPG	Avanti Polar Lipids, Alabama, USA	CAS Number 26853-31-6
1-palmitoyl-2-oleoyl-sn-glycero-3-phospho-(1'-rac-glycerol) (sodium salt); POPC	Avanti Polar Lipids, Alabama, USA	CAS Number 268550-95-4
N,N-Dimethyldodecylamine N-oxide	Sigma Aldrich	CAS Number 1643-20-5
Human insulin	Sigma Aldrich	CAS Number: 11061-68-0
Bovine milk β-lactoglobulin	Sigma Aldrich	CAS Number: 9045-23-2

**Deposited data**		

Raw and analyzed data	This paper	N/A

**Software and algorithms**		

Igor Pro	WaveMetrics	https://www.wavemetrics.com/
Origin	OriginLab	https://www.originlab.com/
Gwyddion software	(Nečas and Klapetek 2012)	http://gwyddion.net/
Topspin v. 3.2 and v. 4.0.7	Bruker	https://www.bruker.com/
OPUS 5.5	Bruker	https://www.bruker.com/
MassLynx	Waters Corporation	https://www.waters.com/nextgen/us/en.html
mMass	(Strohalm et al., 2008)	http://www.mmass.org/

### Resource availability

#### Lead contact

Further information and requests for resources and reagents should be directed to and will be fulfilled by the lead contact, Cecilia Mörman (cecilia.wallin@dbb.su.se or cecilia.morman@outlook.com).

#### Materials availability

This study did not generate new unique reagents.

#### Data and code availability

• All data reported in this paper will be shared by the lead contact upon request.
• This paper does not report original code.
• Any additional information required to reanalyze the data reported in this paper is available from the lead contact upon request.

### Method details

#### Sample preparation

Recombinant Amyloid-β_1-42_ (Aβ_42_) wild type peptide (*rPeptide*, Watkinsville, USA) with the primary amino acid sequence DAEFR_5_HDSGY_10_EVHHQ_15_KLVFF_20_AEDVG_25_SNKGA_30_IIGLM_35_VGGVV_40_IA was pre-treated, if not otherwise stated for each method, with HFIP from the supplier and then dissolved in 10 mM NaOH and sonicated for 5 min in an ice-water bath. Recombinant Amyloid-β_1-40_ (Aβ_40_) wild type peptide (*AlexoTech AB*, Umeå, Sweden), lacking the last two C-terminal residues (I41 and A42) of the Aβ_42_ isoform, was dissolved by adding NaOH to a final concentration of 10 mM and sonicated for 1-3 min in an ice-water bath. A final concentration of 10-20 mM sodium phosphate buffer (pH 7.4 for Aβ_40_ and pH 8 for Aβ_42_) was used for most measurements, prepared by mixing of the samples in NaOH with a slightly higher concentration of sodium phosphate buffer to reach a final buffer concentration of 10-20 mM. The pH of the samples was measured with a newly calibrated electrode pH-meter. MOPS buffer and ammonium acetate were used for some experiments, buffer compounds which do not change the general aggregation behavior of Aβ compared to sodium phosphate buffer. Peptide concentration was determined using a NanoDrop spectrophotometer for the single intrinsic tyrosine residue absorbance at 280 nm with an extinction coefficient of 1490 M^-1^ cm^-1^. Uniformly isotopically ^15^N-labelled Aβ_40_ peptides (*AlexoTech AB*, Umeå, Sweden) were used for the NMR measurements.

Synthetic NCAM1_1-19_-mPrP_23-28_ (NCAM-PrP) peptide construct (*PolyPeptide Group*, Strasbourg, France) with the primary amino acid sequence of MLRTK_5_DLIWT_10_LFFLG_15_TAVSK_20_KRPKP_25_-NH_2_ was dissolved in milliQ-H_2_O to the desired concentration. The peptide concentration was determined spectrophotometrically at 280 nm using tryptophan absorbance (extinction coefficient of 5690 M^-1^ cm^-1^). Signal peptide predictions were performed by the SignalP 5.0 method Almagro Armenteros et al., 2019, Nielsen et al., 1997. Intrinsic solubility (IS) score was calculated according to the CamSol method (Sormanni et al., 2015).

#### Fluorescence spectroscopy – calcein leakage assay using large unilamellar vesicles LUVs

The LUVs were prepared by dissolving phospholipids (zwitterionic POPC and negatively charged POPG, *Avanti Polar Lipids*, Alabaster, Alabama, USA), in chloroform solution. The solvent was subsequently evaporated under a gentle stream of nitrogen (N_2_).The obtained lipid film was resuspended in 50 mM potassium phosphate buffer (KPi) containing 55 mM calcein, pH 7.4. The lipid solution was then vortexed for 10 min, frozen and thawed in liquid N_2_ five times to obtain unilamellar vesicles. The lipid solution was passed 21 times through two polycarbonate filters to obtain vesicles with a 100 nm diameter. The excessive calcein solution was removed by filtering the solution through previously equilibrated PD-10 columns. The size of the LUVs was verified using dynamic light scattering (DLS).

The release of entrapped calcein from the LUVs was measured as an increase in the fluorescence intensity with the Horiba Jobin Yvon Spectrofluorometer (*Longjumeau*, France). A single measurement per condition was included for this measurement series. The excitation wavelength was set to 490 nm, while the emission fluorescence was measured in the range of 500-550 nm. The release of calcein from 100 μM LUVs upon addition of different concentrations of the NCAM-PrP (0.5 μM and 1 μM) and Aβ (1 μM) was measured over time at +5°C. 50 μl of Triton X-100 was added to break the vesicles by inducing 100% leakage. The maximum fluorescence intensity measured at 516 nm was extracted from spectra obtained for all the samples. The data was analyzed by the Origin software. The release of calcein was calculated according to the Equation 1 (Madani and Gräslund 2015).

(Equation 1) $$\text{\% leakage}=\frac{\left(F-F_{0}\right)}{\left(F_{t}-F_{0}\right)}x\;100\%$$

where F_0_ and F_t_ are the fluorescence intensities at 516 nm observed without addition of peptide or the maximal fluorescence intensity obtained after addition of Triton X-100, respectively. F is the fluorescence intensity at 516 nm in the presence of the peptide.

#### Fluorescence spectroscopy – Thioflavin T (ThT) amyloid aggregation kinetics

Prior to all ThT amyloid aggregation kinetics experiments, the Aβ peptides, after sonication in an ice water bath for 1-3 min in 10 mM NaOH, were subject to one further purification step using size exclusion chromatography (SEC) using a Superdex 75 10/300 GL column (*GE Healthcare*, USA), to remove aggregated peptides from the monomeric peptides according to previously published protocols (Wallin et al., 2020). Prepared Aβ_40_ (12 μM) and Aβ_42_ peptide (5 μM) solutions were supplemented with ThT (40 μM for Aβ_40_ and 10 μM for Aβ_42_) in 20 mM phosphate buffer (pH 7.4 for Aβ_40_ and pH 8 for Aβ_42_) and used for the aggregation kinetics measurements. ThT was excited at 440 nm and emission was measured every 2 min at 480 nm in a 96-well plate reader at a 100 μl sample volume (*FLUOstar Omega*, BMG LABTECH, Germany) under quiescent conditions. Three to five replicates per condition were measured. The data was analyzed with Igor Pro. The phenomenological aggregation parameters were determined from sigmoidal curve-fitting according to Equation 2 (Hellstrand et al., 2010).

(Equation 2) $$F\left(t\right)=F_{0}+\;\frac{A}{1+e^{rmax\left(\tau_{½}-t\right)}}$$

where F_0_ is the fluorescence intensity baseline, A is the amplitude, r_max_ represents the maximum growth rate and τ_½_ is the aggregation half time, referred to as the time when 50% of the monomer concentration is depleted.

##### Salt dependence

To study the influence of an increase in ionic strength on the aggregation kinetics, samples containing Aβ_40_ and Aβ_42_ were incubated over time in the presence of 150 mM NaF and NCAM-PrP peptides. The ratio between concentration of the Aβ and NCAM-PrP samples was 1:1.

##### Seeding experiments

Seeding experiments were performed with samples containing 5 μM Aβ_42_ peptides and a fixed concentration of pre-formed Aβ_42_ seeds, in 20 mM MOPS buffer pH 7.8. The samples were incubated over time with 5 μM NCAM-PrP and 10% Aβ_42_ pre-formed fibrils. The pre-formed seeds were prepared upon incubation of monomeric Aβ_42_ peptides for 24 h at +37°C to the fibrillar state. The fibrils were homogenized using sonication. The concentration of seeds was determined based on the monomer concentration.

#### Solid state atomic force microscopy (AFM)

The AFM images were recorded using a Scan Asyst, *Bruker Corp.*, USA operating in tapping mode, at room temperature in air. Sampling rate ranged from 2.0 to 3.5 Hz, while the resolution varied from 256 x 256 to 512 x 512 pixels. Samples containing 5 μM Aβ_42_ peptide and NCAM-PrP in a 1:1 ratio from the end of a fibrillization kinetic experiment were used for AFM imaging. Samples of 50 μl were diluted in 50 μl of milliQ-H_2_O and applied on freshly prepared mica substrates (*Electron Microscopy Sciences*, Hatfield, USA) which were subsequently washed three times with milliQ-H_2_O and dried at room temperature overnight. The AFM images were processed using the Gwyddion software (Nečas and Klapetek 2012).

#### Circular dichroism (CD) spectroscopy

CD spectra were recorded between 190-250 nm (bandwidth of 1.0 nm) using a Chirascan CD spectrometer (*Applied Photophysics*, Leatherhead, U.K.). A quartz cuvette with a 4 mm path length and with a step size of 1.0 nm and a time per point of 4 s were used for recording the spectra. Control spectra were recorded for samples containing only Aβ_40_ (10 μM), Aβ_42_ (5 μM) and NCAM-PrP (5 μM) in 10 mM sodium phosphate buffer, pH 7.4 (Aβ_40_) and pH 8 (Aβ_42_). The NCAM-PrP was titrated to the cuvette containing Aβ peptides in the designed ratios of 1:2 and 1:1. Α single spectrum was recorded for each titration as well as a 4 h long kinetic experiment at a constant temperature of +37°C with data points recorded every 5 min and with continuous stirring using a small magnetic bar.

#### Nuclear magnetic resonance (NMR)

A 700 MHz Bruker Avance NMR spectrometer equipped with a triple resonance cryogenic probe was used for all NMR measurements. Spectra were referenced to trimethylsilyl propanoic acid (TSP). The contribution of TSP to the data was controlled for, with negligible impact on the interaction between Aβ and NCAM-PrP, as well as for the aggregation. Evolution periods are 0.12 s and 34.7 ms in F2 and F1 dimensions. Line broadening is SIN square shifted 90° and exponential 0.3 Hz. The residual assignments are references to previous work (Roche et al., 2016; Danielsson et al., 2007; Jarvet et al., 2007). The data was processed using the Topspin 3.2 and Topspin 4.0.7 software.

To study the monomeric NCAM-PrP interaction with monomeric Aβ_40_ peptides over time, samples of 20 μM uniformly labelled ^15^N-Aβ_40_ peptides in 20 mM MOPS buffer pH 7.35 were incubated for 48 h at +37°C and shaking conditions (200 rpm) in the absence and presence of 5 μM NCAM-PrP. ^1^H-^15^N-HSQC spectra were recorded at +5°C before and after the incubation. The temperature for measuring the HSQC spectra was chosen based on optimal signal intensity and to avoid further aggregation. The interaction between monomeric Aβ peptides and NCAM-PrP was also studied in the presence of SDS micelles. The sample consisted of 84 μM uniformly ^15^N-labelled Aβ_40_ peptides and 50 mM SDS-d25 (>critical micelle concentration (CMC)) in 20 mM NaP buffer, pH 7.3. The ^1^H-^15^N-HSQC spectra with SDS micelles were recorded at +25°C. Monomeric NCAM-PrP peptides were titrated onto the ^15^N-Aβ_40_ sample to ratios of 1:2, 1:1, and 2:1.

#### Aβ exchange dynamics in presence of NCAM-PrP

^15^N-Carr-Purcell-Meiboom-Gill (CPMG) relaxation dispersion experiments (Carr and Purcell 1954; Meiboom and Gill 1958; Tollinger et al., 2001; Wallin et al., 2020) were performed to study exchange dynamics on the ms-μs timescale of Aβ_40_ upon addition of NCAM-PrP peptide. The relaxation dispersion experiments were performed on the sample containing 70 μM ^15^N-Aβ_40_ peptides, 30 μM NCAM-PrP in 50 mM NaP buffer pH 7.3. The total relaxation delay $\tau_{CP}$ was set to 120 ms and refocusing pulses in the frequency range between υ_CPMG_ = 25 to 750 Hz were used.

#### Fourier transform infrared (FTIR) spectroscopy

All samples for infrared (IR) spectroscopy were prepared in D_2_O-based 20 mM sodium phosphate buffer (pD 7.4). The IR measurements were made for 200 μM Aβ_40_ solution (after lowering the pD from the alkaline in 10 mM NaOD solution to 7.4 for the phosphate buffer), 200 μM NCAM-PrP, 200 μM Aβ_40_ with 100 μM NCAM-PrP and 200 μM Aβ_40_ with 200 μM NCAM-PrP.

Transmission FTIR spectra were recorded on a Tensor 37 spectrophotometer (*Bruker*, Germany). The instrument was equipped with an HgCdTe detector continuously purged with dry air. For assembling the IR cuvette, 7-8 μl of the sample was loaded on a flat CaF_2_ window and a second window was added with a 50 μm plastic spacer in between. The assembled cuvette was mounted into the instrument’s sample shuttle and a grid was used in the reference spectrum position to reduce the light intensity in the reference measurement (Baldassarre and Barth 2014). The IR spectra were recorded 20 min after closing the chamber’s lid to avoid interference from water vapor. The spectra were recorded at room temperature, at a resolution of 2 cm^-1^, with a 3.5 mm aperture and using two filters to restrict the light intensity to the spectral range of interest. A Ge filter blocked the light above 2300 cm^-1^ and a cellulose filter reduced the light below 1500 cm^-1^ (Baldassarre and Barth 2014). 100 scans were taken for each spectrum. OPUS 5.5 software was used for acquisition and analysis of the spectra.

#### Mass spectrometry (MS)

For native mass spectrometry (MS) measurements lyophilized Aβ_42_ was dissolved in 6 M guanidine hydrochloride. Lyophilized NCAM-PrP was dissolved in milliQ-H_2_O. Both peptides were subjected to purification using SEC on a Superdex 75 10/300 GL column in 20 mM ammonium acetate (pH 8.3). The NCAM-PrP peptide was subsequently buffer exchanged overnight at +4°C into milliQ-H_2_O by dialysis in a mini dialysis tube with a 1 kDa mass cut-off (*GE Healthcare*). Peptide concentrations were determined spectroscopically using a Nanophotometer (*Implen*). Samples for native ion mobility mass spectrometry were prepared in 100% aqueous ammonium acetate solutions (*Invitrogen*), with and without N,N-Dimethyldodecylamine N-oxide (LDAO) (*Sigma Aldrich*) at 2 x CMC (4 mM).

Native ion mobility mass spectrometry was performed on a Synapt G2S hybrid traveling wave ion mobility-QToF mass spectrometer (*Waters*). Samples were introduced in metal-coated borosilicate emitters (*Thermo Scientific*) using a nanoelectrospray ion source operating in positive ion mode. Instrument parameters were as follows: Capillary voltage 1.5 kV, Sampling cone voltage 50 V, Source offset 80 V, Trap gas flow 10 mL/min, Helium gas flow 100 mL/min, IM gas flow 50 mL/min, IM wave velocity 800 m/s, IM wave height 30 V. A Trap voltage of 50 V was used for dissociation of micelle-bound species; otherwise, a Trap voltage of 5 V was used. Traveling wave ion mobility data was converted into collision cross section (CCS) values by calibration, as has been described previously (Ruotolo et al., 2008), in the following way: Experimental drift time values were measured for calibrant proteins of known CCS. Human insulin (*Sigma Aldrich*) and bovine milk β-lactoglobulin (*Sigma Aldrich*) were used as calibrants. The insulin monomer (+3, +4), dimer (+5, +6) and hexamer (+9, +10, +11) ions, and β-lactoglobulin monomer (+7, +8, +9) and dimer (+11, +12, +13) ions were used for calibration, as they span the m/z range observed for the analytes in this study. Reference CCS values for these calibrants, previously determined on drift tube ion mobility devices, were obtained from literature (Bush et al., 2010; Salbo et al., 2012). A calibration curve that relates the experimental traveling wave ion drift times to CCS was constructed. The data was analyzed using MassLynx (*Waters Corporation*) and mMass (Strohalm et al., 2008).

### Quantification and statistical analysis

The statistical details of experiments can be found in Method details and in the figure legends where applicable. The software used for analysis are mentioned for each method in the Method details.

### Additional resources

No further resources not already mentioned in the manuscript have provided significant additional information.
