# Supplementary material for: The amyloid-inhibiting NCAM-PrP peptide targets Aβ peptide aggregation in membrane-mimetic environments
Source: iScience. 2021 Jul 10;24(8):102852. doi: 10.1016/j.isci.2021.102852 (PMC8340127; doi:10.1016/j.isci.2021.102852)
Supplement: Document S1. Figures S1–S11 [file mmc1.pdf]

iScience, Volume *24*

Supplemental information

The amyloid-inhibiting NCAM-PrP peptide targets Aβ peptide aggregation in membrane-mimetic environments

Sylwia Król, Nicklas Österlund, Faraz Vosough, Jüri Jarvet, Sebastian Wärmländer, Andreas Barth, Leopold L. Ilag, Mazin Magzoub, Astrid Gräslund, and Cecilia Mörman

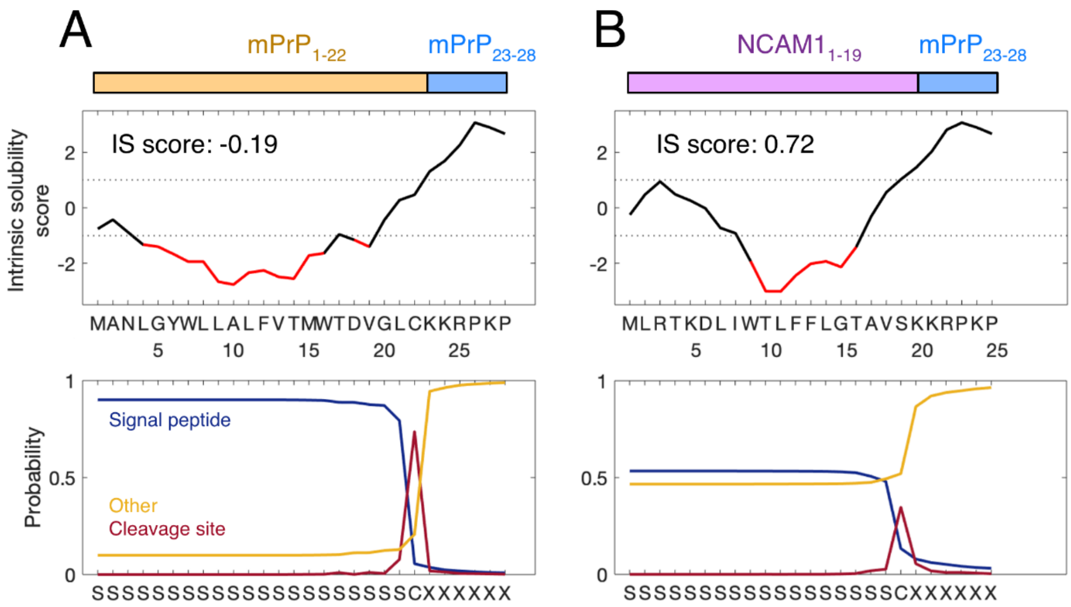

Figure S1. Predicted properties for the mPrP$_{1\text{-}28}$ (A) and NCAM-PrP (B) peptide constructs, related to Figure 1 and Introduction.

The top panels show the calculated intrinsic solubility (IS) score according to the CamSol method (Sormanni et al. 2015). *Regions with an IS score >1 are considered to be highly soluble while regions with an IS score < 1 are considered to be poorly soluble. Poorly soluble segments are highlighted in red. An overall IS score is given for the two peptides, illustrating the increased water solubility of the NCAM-PrP construct used in this study compared to the original mPrP-derived construct. The bottom panels show eukaryotic signal peptide predictions by the SignalP 5.0 method* (Nielsen et al. 1997; Almagro Armenteros et al. 2019). *The predicted propability that the sequence is a signal peptide is plotted in blue, the predicted probability that the sequence is another type of sequence is plotted in yellow, and the predicted probability for a signal peptidase cleavage site is plotted in red. It can be seen that the signal peptidase is predicted to cleave N-terminal to the mPrP$_{23\text{-}28}$ segment in both cases (predicted cleavage site is marked by 'C' in the sequence). Both mPrP1-22 and NCAM1$_{1\text{-}19}$ are recognized by SignalP 5.0 as eukaryotic signal peptides which target secretion (marked by 'S' in the sequence).*

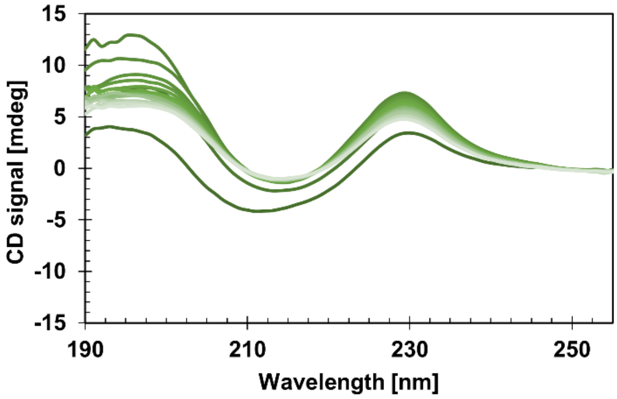

Figure S2. CD spectra of NCAM-PrP over time, related to Figure 2.

20 µM NCAM-PrP was incubated over time (0-4 h) in 10 mM NaP buffer pH 7.3 at +37 °C with constant stirring using a small magnet. The spectrum recorded at time zero is dark green, followed by spectra with subsequent lighter colour reaching 4 h.

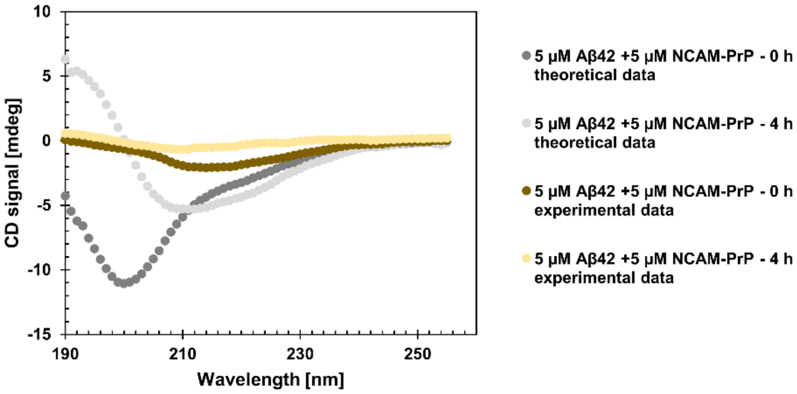

Figure S3. Comparison of CD-signal at time zero and 4 h for theoretical and experimental spectra with Aβ_{42} and NCAM-PrP, related to Figure 2.

Spectra from Figure 2C and D in the main manuscript are here visualized for a clear comparison. 5 µM Aβ_{42} and 5 µM NCAM-PrP in 10 mM NaP buffer pH 7.3 at +37 °C with constant stirring using a small magnet was used.

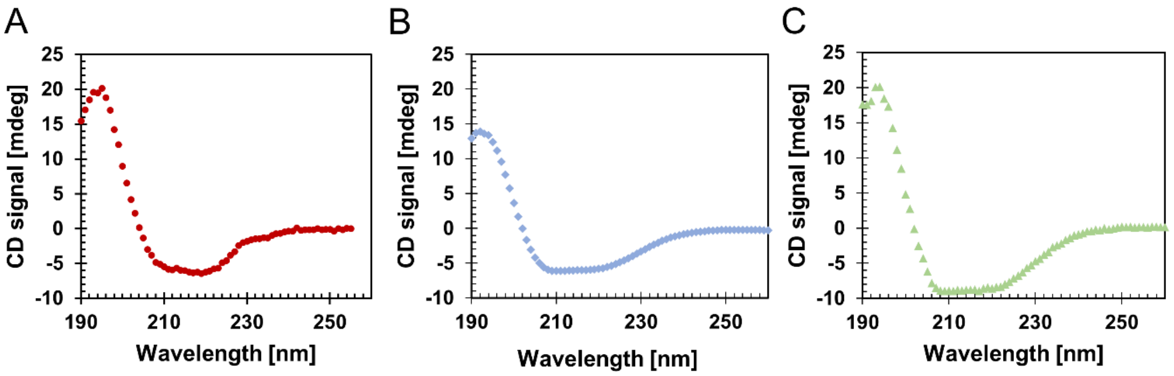

Figure S4. CD spectra of NCAM-PrP and Aβ_{42} + NCAM-PrP in the presence of SDS micelles, related to Figures 2 and 5D.

***(A)** CD spectrum of 5 µM NCAM-PrP in 10 mM NaP buffer pH 7.4 after addition of 50 mM SDS (>CMC). After the kinetic experiment, of 5 µM Aβ_{42} peptides alone **(B)** in Figure 2A in the main manuscript and in the presence of 2.5 µM NCAM-PrP **(C)** in Figure 2E, 50 mM SDS was added and a CD spectrum was recorded.*

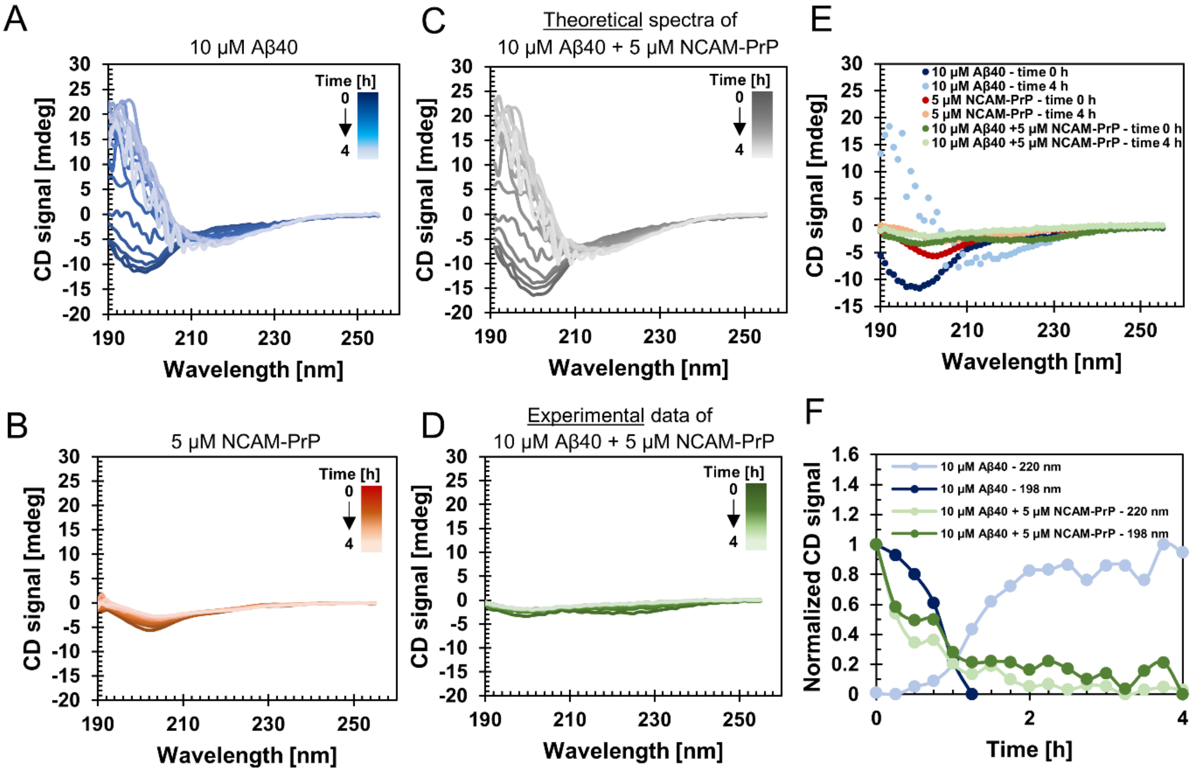

Figure S5. CD spectroscopy to study the secondary structures of $A\beta_{40}$ and NCAM-PrP peptides over time, related to Figure 2.
(A) *10μM $A\beta_{40}$ peptides were incubated in 10 mM NaP buffer pH 7.3 at +37 °C during magnetic stirring.* ***(B)*** *Incubation of 5 μM NCAM-PrP peptides in 10 mM NaP buffer pH 7.3 at +37 °C with magnetic stirring. A theoretical spectra presentation of the spectra in* ***(A)*** *and* ***(B)*** *(added at each time point) is presented in* ***(C)****. For clarification purposes, the first and the last spectra in* ***(A, B, D)*** *are presented together in* ***(E)****. In* ***(F)*** *are the first and last spectra in* ***(A, D)*** *normalized and presented together.*

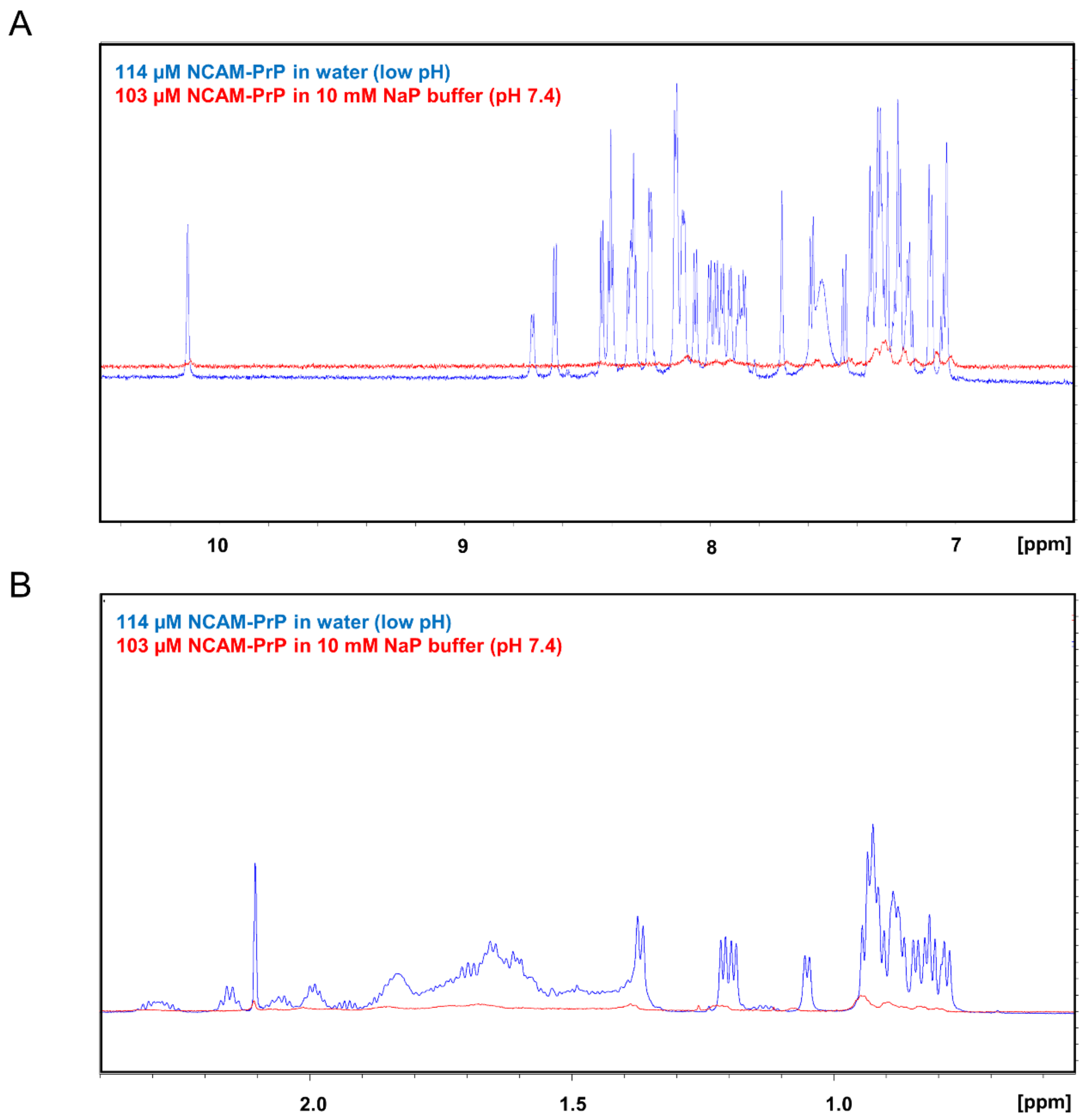

Figure S6. Characterization of NCAM-PrP peptide in water and in buffer, related to Figure 5. *1D NMR spectra of NCAM-PrP peptides in water and in a buffered solution, the amide region is presented in* ***(A)*** *and the methyl region in* ***(B)****. 114 µM NCAM-PrP peptides in water at low pH is shown in blue and 103 µM NCAM-PrP peptides in 10 mM NaP buffer pH 7.4 is presented in red.*

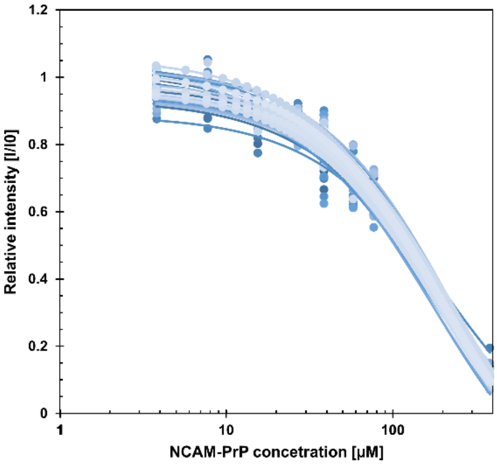

Figure S7. Global fit analysis of 2D NMR ^{1}H-^{15}N-HSQC titration data, related to Figure 5. *NCAM-PrP was titrated onto a 77 µM monomeric ^{15}N-Aβ_{40} peptides in 20 mM NaP buffer pH 7.3. The spectra were recorded at 278 K. The apparent dissociation constant (K_D^{app}) was determined by a global fit analysis of the titration data was performed using the equation* (Tiiman et al. 2016)

$$I = I_0 + \frac{I_\infty - I_0}{2\cdot[A\beta]}\cdot\left(K_D^{app} + [NCAM - PrP] + [A\beta] - \sqrt{\left(K_D^{app} + [NCAM - PrP] + [A\beta]\right)^2 - 4\cdot[NCAM - PrP]\cdot[A\beta]}\right)$$

, where I_∞ is the intensity upon saturation and I_0 the initial intensity. An overall dissociation constant of the Aβ_{40}:NCAM-PrP complex was determined to 160±120 µM. No correction of buffer contributions was made.

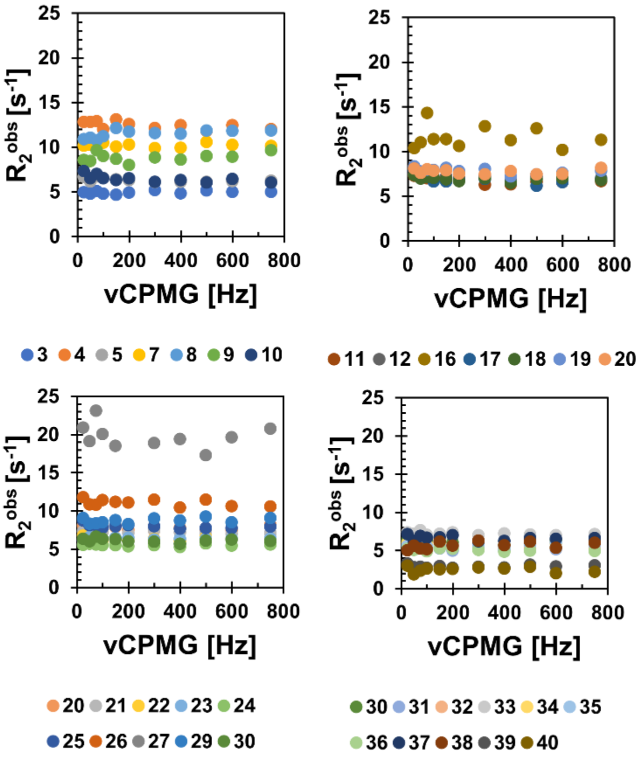

Figure S8. Relaxation dispersion experiments, related to Figure 5.
A sample of 70 µM monomeric ^{15}N-Aβ_{40} peptides in the absence and presence of 30 µM NCAM-PrP in

50 mM NaP buffer pH 7.3 was used for additional NMR experiments. Relaxation dispersion was measured and the relaxation dispersion profiles for the Aβ residues are presented in four plots. The observed transverse relaxation rates R_2^{obs} were calculated from $R_2^{obs} = \frac{1}{\tau_{CP}} ln \frac{I_0}{I}$ where I_0 is the intensity of a cross-peak from a reference experiment where τ_{CP} = 0, i.e. the CPMG pulse block is omitted, and I is the intensity of the cross-peak at a given refocusing frequency. No significant ($p < 0.01$) chemical exchange was observed under these experimental conditions.

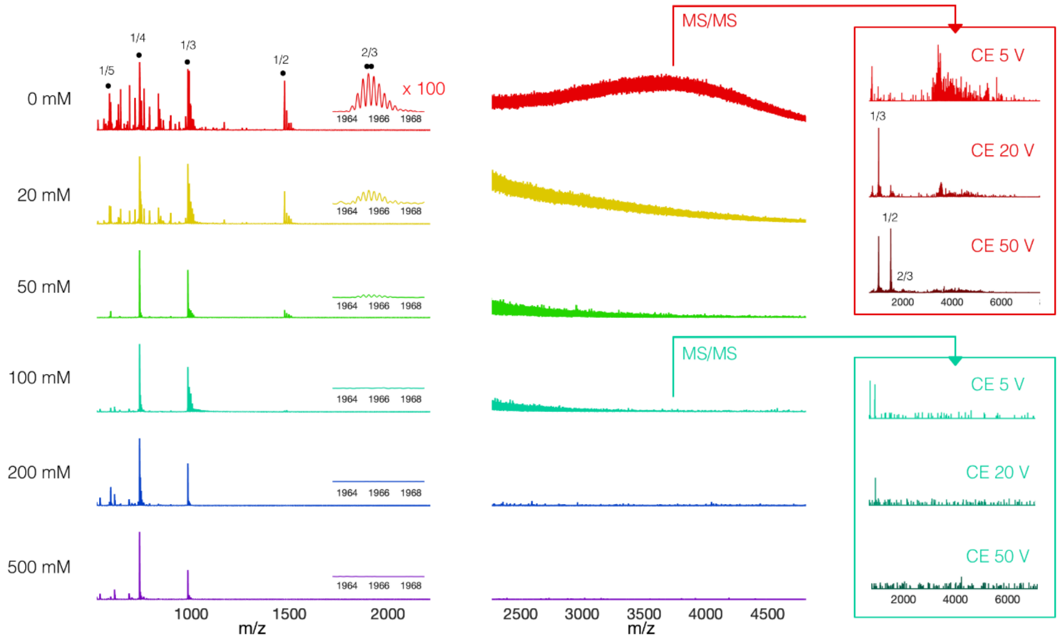

***Figure S9. Mass spectra of 10 µM NCAM-PrP, related to Figure 6,** in varying amount of the volatile salt ammonium acetate at low and high m/z. Red squares indicate NCAM-PrP signals, numbers indicate the oligomer to charge ratio (n/z). The 2/5 dimeric species (+5 charged) is magnified in the insert. Unresolved features are present at high m/z which dissociate into NCAM-PrP monomers upon collisional activation in MS/MS.*

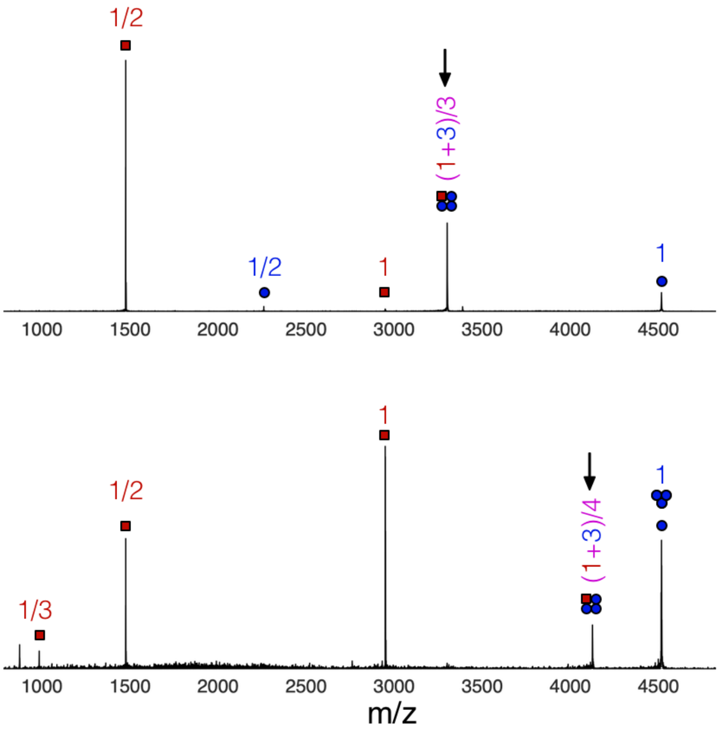

Figure S10. MS/MS of the 1+3 NCAM-PrP/Aβ heterooligomer, related to Figure 6, *at 3+ (top) and 4+ (bottom) charge states. MS/MS confirms that ions consist of an aggregate of NCAM-PrP (red squares) and Aβ (blue circles).*

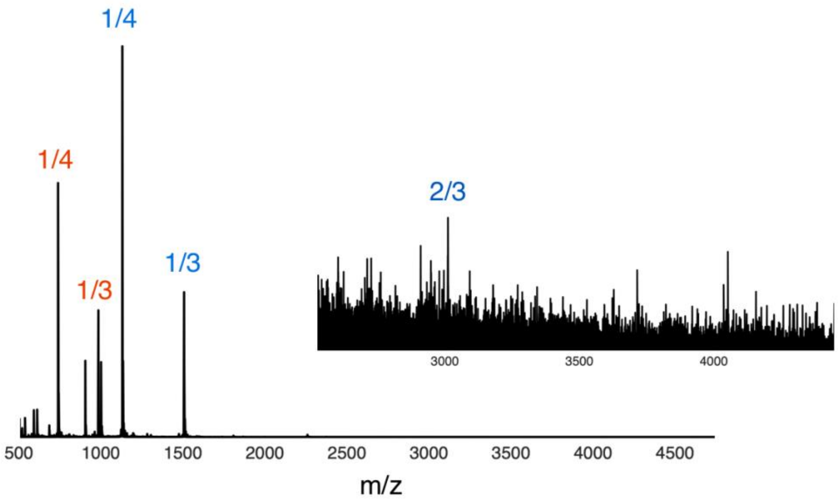

Figure S11. MS analysis of scrambled Aβ(1-42), related to Figure 7, *(KVKGLIDGAHIGDLVYEFMDSNSAIFREGVGAGHVHVAQVEF) under the same conditions as samples in Figure 6A (4 mM LDAO detergent, under collisional activation). Red numbers indicate NCAM-PrP signals while blue numbers indicate Aβ signals. An insert is shown in the region where oligomeric signals are expected.*
